# *In vitro* model of human mammary gland microbial colonization (MAGIC) demonstrates distinctive cytokine response to imbalanced human milk microbiota

**DOI:** 10.1128/spectrum.02369-23

**Published:** 2024-01-30

**Authors:** Primož Treven, Diana Paveljšek, Rok Kostanjšek, Majda Golob, Bojana Bogovič Matijašič, Petra Mohar Lorbeg

**Affiliations:** 1 Department of Animal Science, University of Ljubljana, Biotechnical Faculty, Institute of Dairy Science and Probiotics, Domžale, Slovenia; 2 Department of Biology, University of Ljubljana, Biotechnical Faculty, Chair of Zoology, Ljubljana, Slovenia; 3 University of Ljubljana, Veterinary Faculty, Institute of Microbiology and Parasitology, Ljubljana, Slovenia; Wayne State University, Detroit, Michigan, USA

**Keywords:** human milk microbiota, bacterial adherence, cytokine response

## Abstract

Despite the established concept of the human mammary gland (MG) as a habitat with its own microbiota, the exact mechanism of MG colonization is still elusive and a well-characterized *in vitro* model would reinforce studies of the MG microbiota development. We aimed to establish and characterize an *in vitro* cell model for studying MAmmary Gland mIcrobial Colonization (MAGIC) model. We used the immortalized cell line MCF10A, which expresses the strong polarized phenotype similar to MG ductal epithelium when cultured on a permeable support (Transwell). We analyzed the surface properties of the MAGIC model by gene expression analysis of E-cadherin, tight junction proteins, and mucins and by scanning electron microscopy. To demonstrate the applicability of the model, we tested the adhesion capability of the whole human milk (HM) microbial community and the cellular response of the model when challenged directly with raw HM samples. MCF10A on permeable supports differentiated and formed a tight barrier, by upregulation of CLDN8, MUC1, MUC4, and MUC20 genes. The surface of the model was covered with mucins and morphologically diverse with at least two cell types and two types of microvilli. Cells in the MAGIC model withstood the challenge with heat-treated HM samples and responded differently to the imbalanced HM microbiota by distinctive cytokine response. The microbial profile of the bacteria adhered on the MAGIC model reflected the microbiological profile of the input HM samples. The well-studied MAGIC model could be useful for studies of bacterial attachment to the MG and for *in vitro* studies of biofilm formation and microbiota development.

**IMPORTANCE:**

The MAGIC model may be particularly useful for studies of bacterial attachment to the surface of the mammary ducts and for *in vitro* studies of biofilm formation and the development of the human mammary gland (MG) microbiota. The model is also useful for immunological studies of the interaction between bacteria and MG cells. We obtained pioneering information on which of the bacteria present in the raw human milk (HM) were able to attach to the epithelium treated directly with raw HM, as well as on the effects of bacteria on the MG epithelial cells. The MAGIC cell model also offers new opportunities for research in other areas of MG physiology, such as the effects of bioactive milk components on microbial colonization of the MG, mastitis prevention, and studies of probiotic development. Since resident MG bacteria may be an important factor in breast cancer development, the MAGIC *in vitro* tool also offers new opportunities for cancer research.

## INTRODUCTION

In recent decades, the use of advanced culture-dependent and independent techniques has shown that breast milk is a complex ecosystem containing representatives of various bacterial genera such as *Streptococcus*, *Staphylococcus*, *Weissella*, *Clostridium*, *Serratia*, *Cutibacterium* (formerly known as *Propionibacterium*), bifidobacteria, and lactic acid bacteria (1, 2). The process of microbial colonization of the mammary gland (MG) is still debatable, and most likely, the bacterial biofilm is formed in MG ducts (3). Research in the field of MG microbiota is mainly focused on microbial dysbiosis and mastitis in cattle and is mainly performed *in vivo* directly in bovine MGs (4), in the mouse mastitis model (5), or *in vitro* on bovine mammary cell lines, such as MAC-T (6). Human MG cell-based models are mainly used for studies on basic MG development, morphogenesis, carcinogenesis, milk production, and immunological responses (7). For the study of microbial attachment and colonization, there is a need for a well-characterized human cell-culture model of the MG epithelial surface.

Expression of mucins on the epithelial surface is a key feature of all mucosal surfaces. Although human MG is not considered a classical mucosal organ, it exhibits some characteristics which are common to mucosal tissues such as strong mucosal immune programs and mucin expression on the surface of epithelial cells (8). Expression of mucins on the cellular surface is an important feature which helps to protect the epithelial surface on the one hand and enables bacterial adherence on the other hand (9).

The MCF10A cell line is one of the most widely used human MG epithelial cell lines (10). It was derived from benign mammary tissue and spontaneously immortalized (11). MCF10A cells cultured on porous cell culture membranes (PCCM) assemble tight junctions and thus develop a low conductivity barrier (12–14). The cells express Mucin 1 and differentiate to a stage similar to ductal epithelium. Moreover, MCF10A cells exhibited multipotent phenotypic differentiation into layers expressing basal (CD10^+^, MUC1^−^) and luminal (CD10^−^, MUC1^+^) markers with at least two distinct cell phenotypes (12). The epithelial nature of MCF10A was also confirmed by the fact that the media composition of the lower chamber (basolateral) has a major impact on the maintenance of the membrane barrier function (12). The system of MCF10A cells grown on PCCM appears to be useful for simulation of MG microbial colonization (MAGIC model) on ductal epithelium, similar to cell-based models of the intestinal tract commonly used in various *in vitro* studies such as bacterial adherence or cytotoxicity assays (15, 16).

Thus, our goal was to establish and optimize the growth of MCF10A cells on PCCM and characterize the surface properties and potential mucin gene expression of the MAGIC model. The second objective was to validate the applicability of the MAGIC model to study the microbial colonization process of the MG. We tested the adhesion capability of the whole human milk (HM) microbial community and cellular response of the model to a direct challenge with HM samples. We demonstrated that the MAGIC model could be applicable for novel studies of microbial development on the mucus-secreting surface of the mammary epithelium and of the influence of pathogenic bacterial strains on bacterial colonization of MG and its inflammatory response.

## RESULTS

### The MAGIC model develops a tight barrier with a morphologically diverse surface, covered with mucins

**Fig 1:**
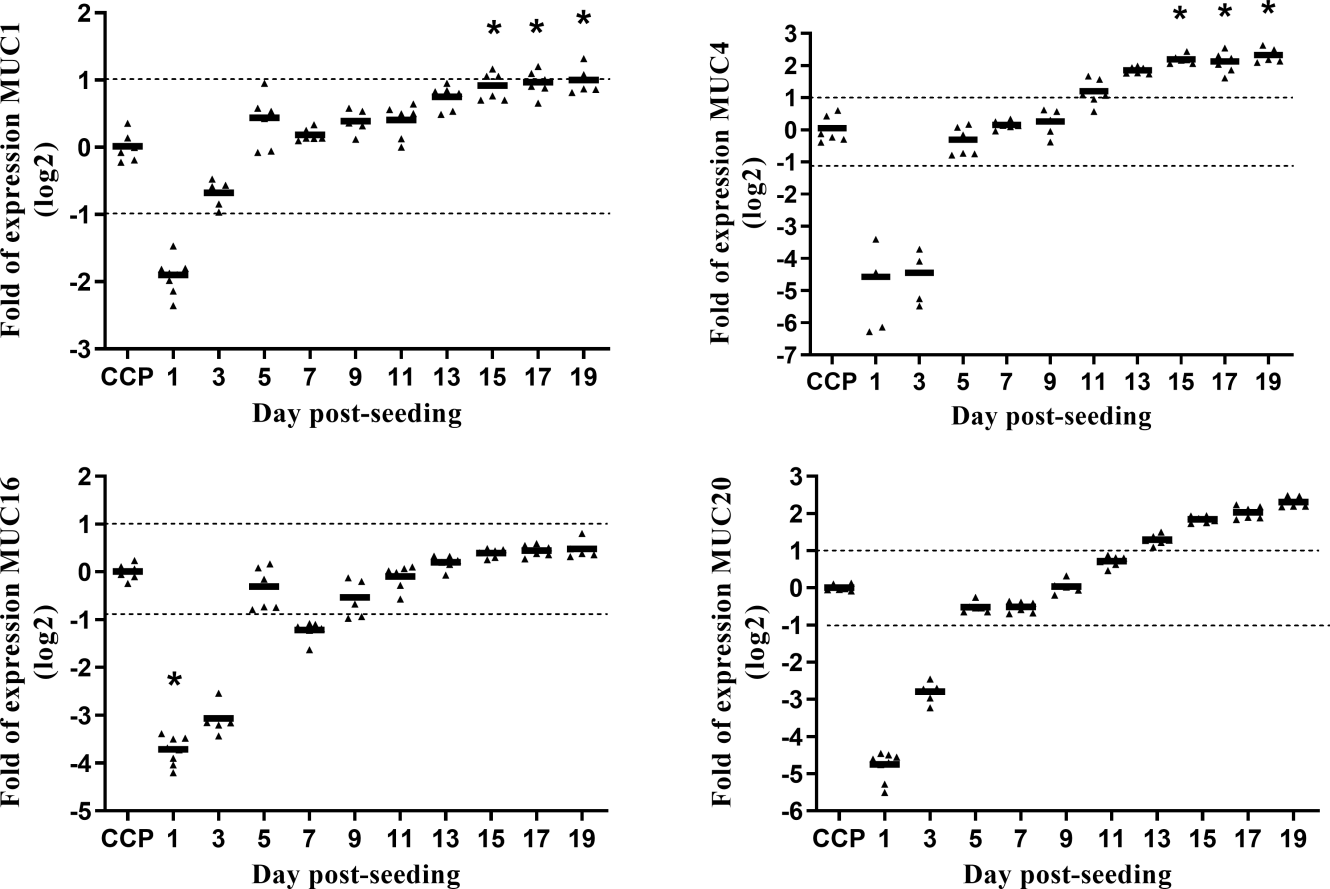
Relative gene expression for mucins during the cell growth on PCCM. Results are shown as normalized gene expression relative to the expression in cells confluent on 48-well CCP. Statistical significance was analyzed with non-parametric one-way analysis of variance(ANOVA) (Kruskal-Wallis statistic) and Dunn’s multiple comparison test with adjustments of *P*-values to account for multiple comparisons; *P < 0.05. The experiment was performed in three independent experiments with two biological and two technical replicates. Dotted line marks twofold change in gene expression.

Cells grown on PCCM reached peak transepithelial electrical resistance (TEER) in 14–17 days after seeding and formed a tight barrier with high TEER (above 1,500 Ω × cm^2^) and low fluorescein isothiocyanate(FITC) influx (Fig. S1B). Growth of MCF10A cells on PCCM was characterized by a slight upregulation (1.6-fold at day 13) of the CLDN1 and upregulation of the CLDN8 gene, with mean expression reaching a significant eightfold upregulation at day 19 compared with the expression in cells on cell culture plates (CCP) (Fig. S2). We observed only a slight increase in the expression of the MUC16 gene (Fig. 1) (1.4-fold at day 19) and a moderate but significant upregulation of the MUC1 gene (twofold at day 19). There was also fivefold increase in the expression of the MUC4 and the MUC20 genes. The upregulation of the MUC20 was close to statistical significance (*P* = 0.06) on day 19 while upregulation of the MUC4 reached significance at day 15. The presence of the mucin layer on the apical side of the epithelium was also confirmed with Alcian blue/PAS staining of cells fixed at day 19 (Fig. S3).

**Fig 2:**
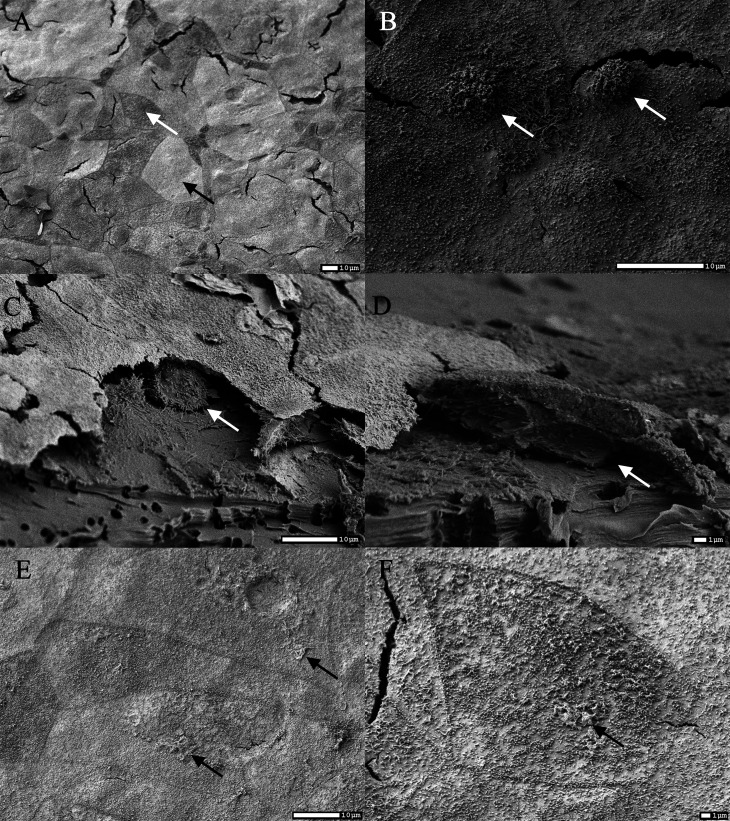
Scanning electron micrograph of the surface of the epithelial cells grown on PCCM. Scale bar and magnification is outlined on each micrograph. Selected figures are representative of each entire sample. (**A**) Cells densely covered with microvilli (white arrow) and cells with almost no microvilli (black arrow). (B) Small flat microvilli (black arrow) and larger round microvilli (white arrow), cells were extensively washed before fixation. (**C and D**) Cross-fracture of the PCCM showing putative third type of the cells (white arrows) squeezed between the filter and the apical cell monolayer. (**E and F**) Cells of different morphologies with the putative residual mucins (black arrows); the cells were fixed without washing.

SEM micrographs of differentiated epithelial cells grown on PCCM showed that the surface of the epithelial cells was morphologically diverse with at least two cell types. One cell type was densely covered with microvilli, whereas on some cells, there were almost no microvilli (Fig. 2A). In addition, we observed two types of microvilli (Fig. 2B)—small flat microvilli and larger round microvilli (Fig. 2B). To check the basolateral side of the cells, dried PCCM were cross-fractured. Fractures revealed a third cell type which was squeezed between the filter and the apical cell layer (Fig. 2C and D). Although the cells were squeezed, they resembled a spherical shape with approx. 10 µm in diameter. The initial protocol for fixation included extensive washing before fixation (twice with HBSS and three times with sodium cacodylate buffer). However, when the cells were fixed without initial washing, putative residual mucins were observed on the surface of the cells (Fig. 2E and F).

### The MAGIC model can withstand the challenge with heat-treated HM samples and respond differently to raw HM samples

**Fig 3:**
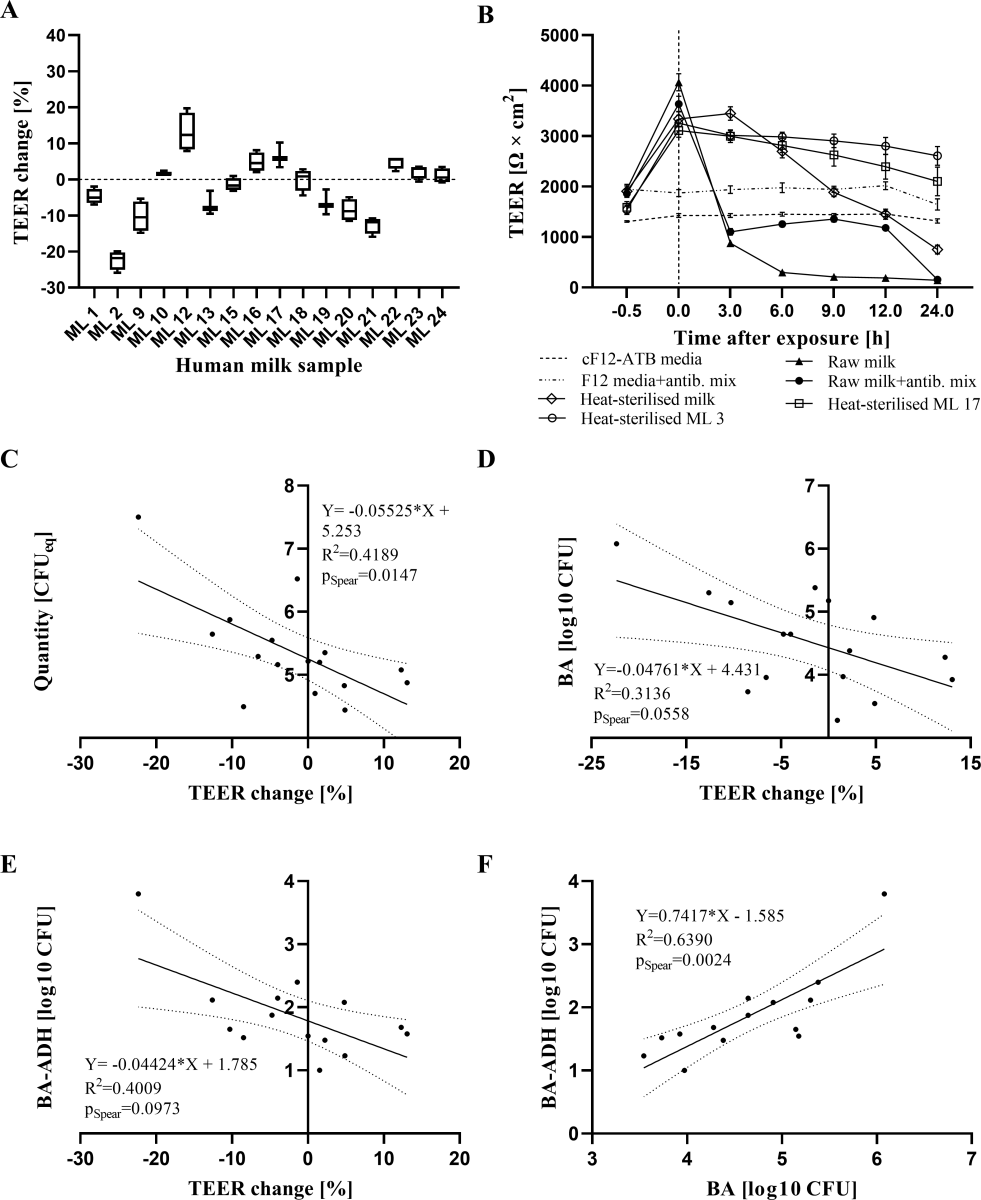
TEER change after the challenge of MAGIC model to HM samples and Spearman rank correlation analysis of the effect of bacterial concentrations on TEER and adhesion. (**A**) TEER development after the challenge of the MCF10A cells with the pooled raw HM, the pooled HM that was heat treated, or the pooled HM treated with the mix of antibiotics and with two randomly selected heat-treated HM samples (ML 3 and ML 17). The experiment was performed in six biological replicates per group. The results are shown as mean ± standard error. (**B**) TEER change after 1-h challenge of the MAGIC model to different HM samples. The experiment was performed in four biological replicates. (**C**) Linear regression between the total bacterial count, determined by qPCR and TEER change. Data of total bacterial count by qPCR were extracted from the publication (17). p_Spear_, *P*-value from Spearman correlation statistics; CFU_eq_, colony forming units equivalents. (**D**) Linear regression between the abundance in BA culture-positive raw HM samples and TEER change. (**E**) Linear regression between the abundance in BA culture-positive raw samples of trypsinized MCF10A cells after the 1-h adhesion assay and TEER change. (**F**) Linear regression between the abundance in BA culture-positive raw samples of trypsinized MCF10A cells after the 1 h adhesion assay and the abundance in BA culture-positive raw HM samples.

To test the applicability of the MAGIC model for studying MG microbial adherence and the development of the MG microbiota, we challenged the model with HM. We challenged the model with the pooled raw HM sample, the heat-treated pooled HM sample, or pooled HM sample treated with mixture of antibiotics (Fig. 3A). In addition, we tested two randomly selected heat-treated HM samples. Immediately after the addition of HM samples, the TEER increased substantially due to the difference in conductivity of the milk compared with the cell culture medium. After 3 h, we observed a significant difference between the pooled raw HM sample and the heat-treated pooled HM sample indicating the deleterious effect of bacterial growth on the differentiated MCF10A cells. The addition of antibiotics slightly mitigated the decrease, so cells maintained TEER above 1,000 Ω × cm^2^ for 12 h. In contrast, heat-treated HM samples sustained the challenge for the first 3 h. However, there was a noticeable difference between pooled HM samples and two randomly selected HM samples at later timepoints, where we noticed a substantial decrease in TEER in the case of pooled HM (Fig. 3A). In addition, we challenged the model with 16 raw HM samples for 1 h. As expected, the levels of TEER differed substantially among different samples (Fig. 3B). For example, TEER decreased by 22% when challenged with ML 2 and increased by 13% with ML 12. The differences in TEER change were negatively correlated with the total bacterial count, determined by quantitative PCR (qPCR) while the bacterial count of HM samples on blood agar (BA) did not reach Spearman correlation significance (Fig. 3C and D). Also, the correlation of abundance in BA-positive samples after the adhesion assay did not reach statistical significance (Fig. 3E).

### The microbial profile of bacteria adhered on the MAGIC model reflected the microbiological profile of the input HM microbiota

**Fig 4:**
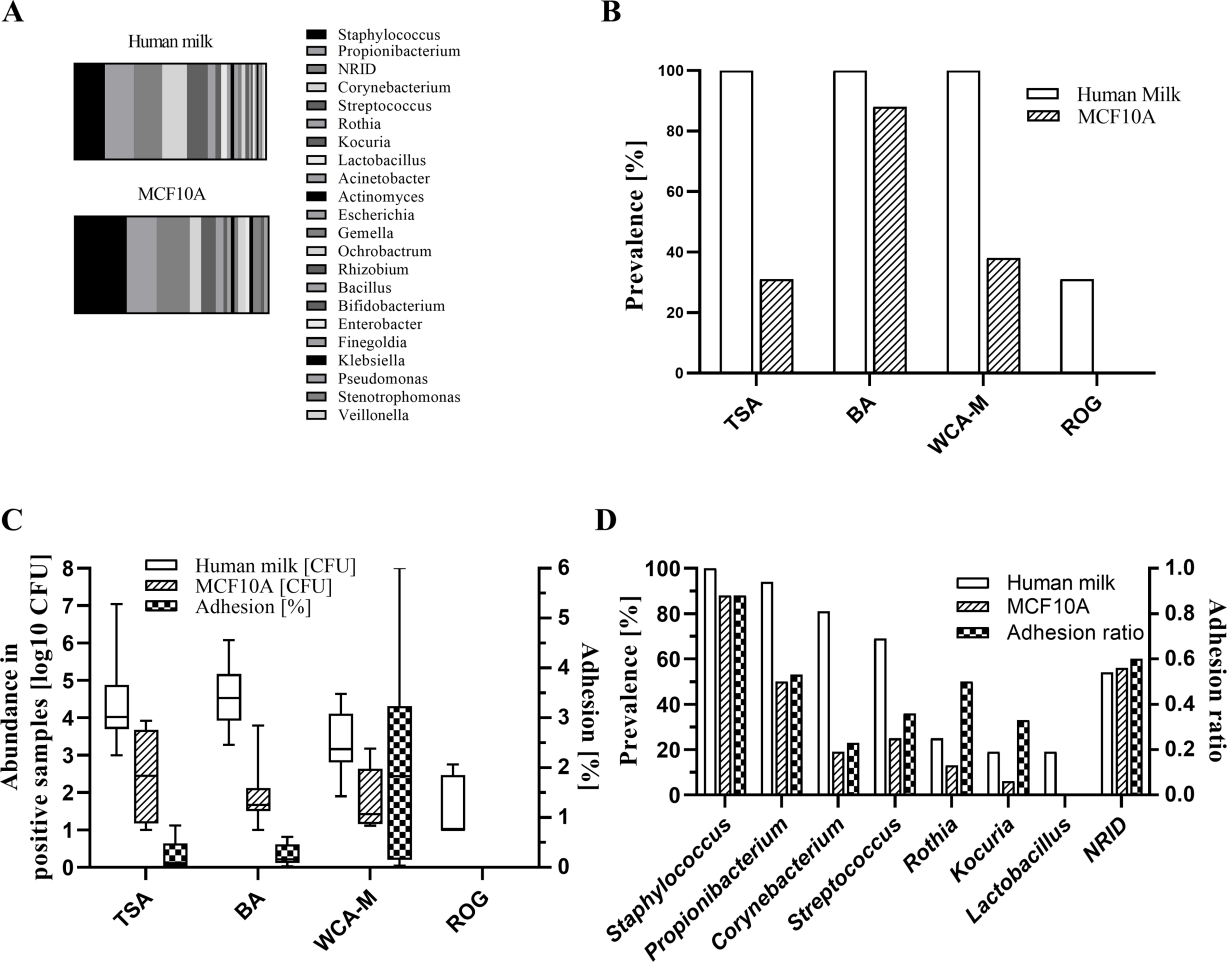
Adhesion ability of bacteria from raw HM to the MAGIC model. (**A**) Comparison of the prevalence of genus-positive samples for raw HM and for trypsinized MCF10A cells after the 1-h adhesion assay. Genera were determined with MALDI-TOF MS. NRID, no reliable identification. (**B**) Prevalence of culture-positive raw HM samples and of trypsinized MCF10A cells after the 1-h adhesion assay. Samples were plated on BA, TSA, WCA-M, and Rogosa (ROG) agar. Plates were incubated at 37°C for 72 h in aerobic (TSA) conditions or anaerobic (BA, WCA-M, and ROG) conditions. (**C**) The abundance in culture-positive raw HM samples and in trypsinized MCF10A cells after the 1-h adhesion assay. Numerical data are presented in Table S5. (**D**) Prevalence of genus-positive samples of raw HM and of trypsinized MCF10A cells after the 1-h adhesion assay. Only genera that were found in more than three HM samples are presented. Complete list of detected genera is available in Table S6.

In general, the microbial profile of bacteria adhered on the surface of cells of the MAGIC model reflected the microbiological profile of the input HM microbiota (Fig. 4A). Also, the bacterial count of HM samples on BA was positively correlated with the number of bacteria detected on BA after the adhesion assay (Fig. 3F). The adhered bacteria were predominantly Gram-positive, facultative anaerobic bacteria (83.6% of positive cases). Gram-negative, aerobic genera such as *Ochrobactrum*, *Stenotrophomonas*, *Acinetobacter*, *Delftia*, and *Neisseria* were detected in only 16.4% of cases which is similar to the ratio observed in human milk samples (12.1%). The prevalence of culture-positive samples after the challenge of the model with different HM samples (Fig. 4B) was significantly higher when samples were plated on BA (88%) compared with tryptic soy agar (TSA) (31%; McNemar’s chi-squared test with continuity correction, *P* = 0.008) or Wilkins-Chalgren Anaerobe agar supplemented with mupirocin (WCA-M) (38%; *P* = 0.013). As expected, the abundance in positive samples was higher when selective WCA-M was used, reaching 2.00% average adhesion compared with 0.21% or 0.24% on TSA or BA, respectively (Fig. 4C).

**Fig 5:**
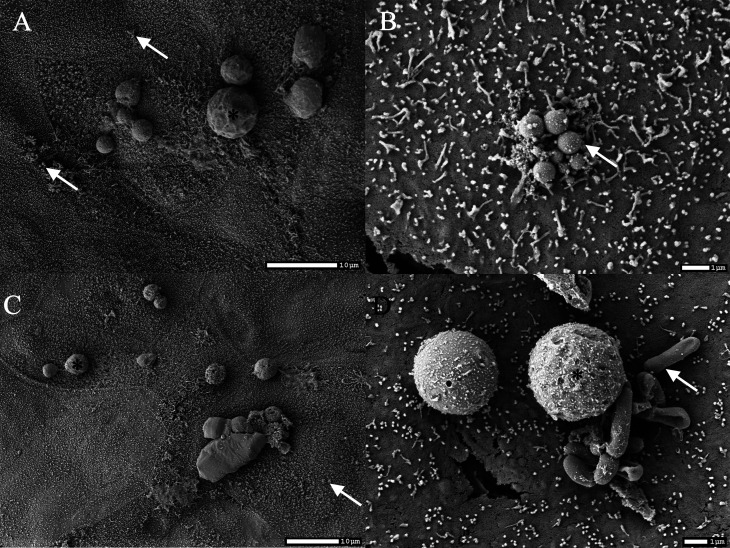
Various bacteria from HM adhered to the MAGIC model. Scanning electron micrograph of the surface of the epithelial cells showing adhered cocci- and bacilli-shaped bacteria (white arrows) after epithelial cells were exposed to raw HM samples for 1 h. Scale bar and magnification are outlined on each micrograph. HM fat globules are marked with black asterisk. Selected figures are representative of the entire sample. (**A and B**) HM sample ML 15, (**C**) HM sample ML 2, and (**D**) HM sample ML 9.

We noticed a considerable difference in the ability of specific genus to adhere to the MAGIC model cells (Fig. 4D). All HM samples tested (16) were positive for *Staphylococcu*s, which also successfully adhered to the model in 87% of samples. Similarly, 94% of the HM samples tested were positive for *Propionibacterium* and they were successful in adherence in 53% of samples. In contrast, the adherence efficiency of *Corynebacterium* was lower. Although 81% of the HM samples tested were positive for *Corynebacterium*, adherence was shown for 23% of the samples only. We observed similar patterns at the species level (Table 1), where adhered *Staphylococcus epidermidis* (*S. epidermidis*) were confirmed for 88*%* of *S. epidermidis*-positive HM samples. In contrast, *Cutibacterium granulosum* and *Streptococcus vestibularis* were not adhered to the model. We confirmed the adhesion of the bacteria using SEM (Fig. 5). Several coccoid and bacilliform bacteria interacted closely with the epithelial cells. In some cases, the cells were in direct contact with the microvilli of the cells (Fig. 5B and C). We also observed damaged bacterial cells that were in close interaction with the epithelial surface (Fig. 5D).

**TABLE 1:** Prevalence of bacterial species in HM samples and their ability to adhere to the MAGIC model after 1-h adhesion assay^*a*^

Species	Human milk	Adhesion on MCF10A	
	No. of positive samples [prevalence (%)]	No. of positive samples [prevalence (%)]	Adherence ratio^*b*^
*Staphylococcus epidermidis*	16 (100.0)	14 (87.5)	0.88
*Cutibacterium^c^ acnes*	15 (93.8)	8 (50.0)	0.53
*Corynebacterium tuberculostearicum*	10 (62.5)	3 (18.8)	0.30
*Staphylococcus hominis*	8 (50.0)	3 (18.8)	0.38
*Streptococcus mitis*	7 (43.8)	2 (12.5)	0.29
*Cutibacterium^c^ granulosum*	4 (25.0)	0 (0.0)	0.00
*Staphylococcus warneri*	4 (25.0)	3 (18.8)	0.75
*Streptococcus oralis*	4 (25.0)	1 (6. 3)	0.25
*Streptococcus salivarius*	4 (25.0)	1 (6.3)	0.25
*Streptococcus vestibularis*	4 (25.0)	0 (0.0)	0.00
*Corynebacterium simulans*	3 (18.8)	0 (0.0)	0.00
*Rothia mucilaginosa*	3 (18.8)	1 (6.3)	0.33
*Streptococcus parasanguinis*	3 (18.8)	0 (0.0)	0.00

^*a*^ Samples were plated on BA, TSA, WCA-M, and ROG agar. Plates were incubated at 37°C for 72 h in aerobic (TSA) conditions or anaerobic (BA, WCA-M, and ROG) conditions. Species of the colonies were determined with MALDI-TOF MS.

^*b*^ Ratio of samples positive for adhesion vs. all culture-positive HM samples.

^*c*^ Former *Propionibacterium.* Only species that were detected in more than three HM samples are listed; the complete list is available in Table S7.

### The MAGIC model demonstrates distinctive cytokine response to imbalanced HM microbiota

**Fig 6:**
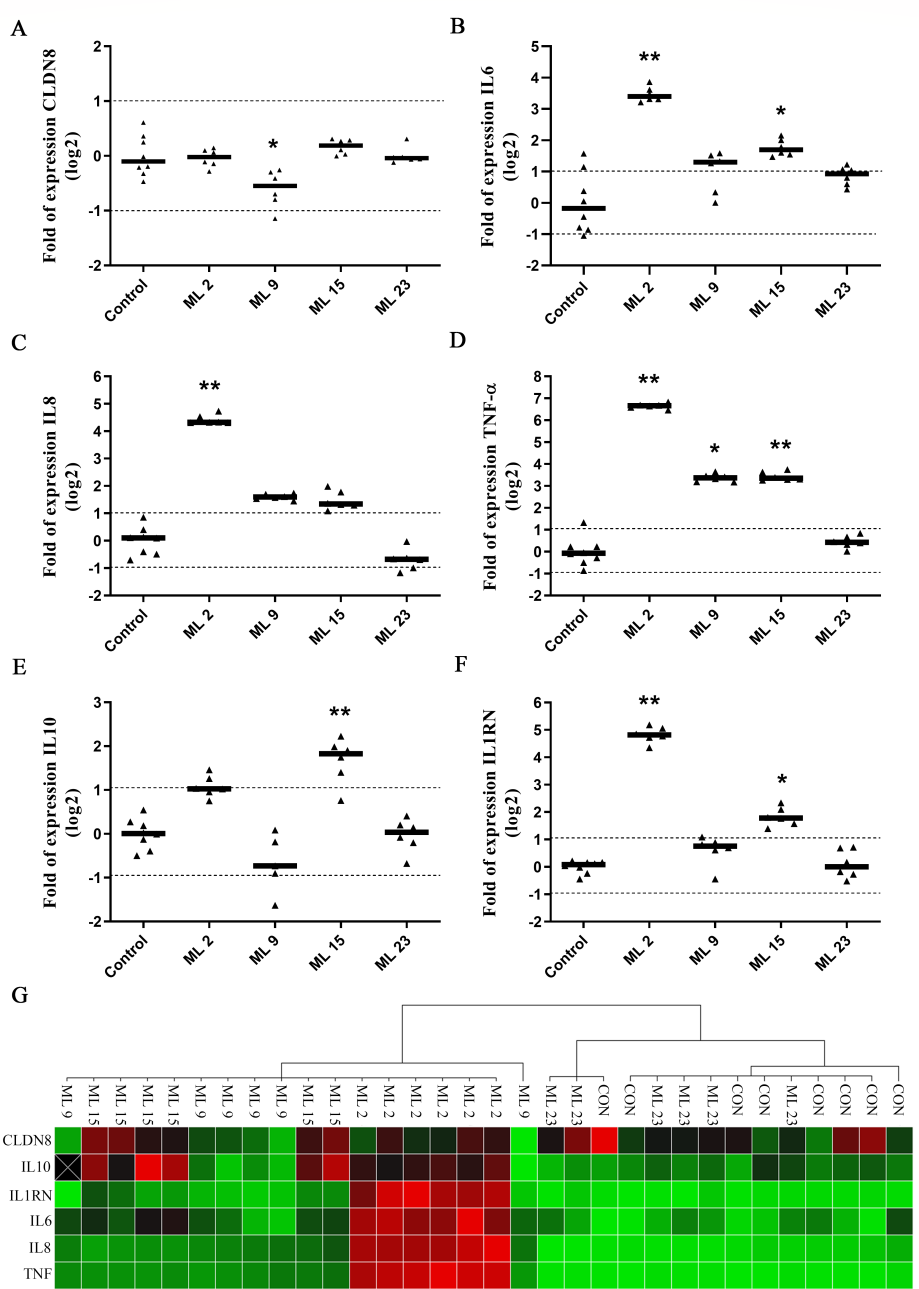
The expression of cytokine-encoding genes after the challenge of MAGIC model with raw HM samples. (A–F) Relative gene expression analysis of genes encoding cytokine proteins after exposure to five raw HM samples for 30 minutes. The results are shown as normalized gene expression relative to control samples—MCF10A cells grown on PCCM. The experiment was performed in three independent experiments with two biological and two technical replicates. Statistical significance was analyzed with non-parametric one-way ANOVA (Kruskal-Wallis statistic) and Dunn’s multiple comparison test with adjustments of *P*-values to account for multiple comparisons; *P < 0.05 and **P < 0.01. (G) The clustergram of normalized gene expression shows HM sample ML 23 clustered together with the control sample, while other samples were clustered separately. The clustergram image depicts relative expression of a sample and a target as follows: red, upregulation; green, downregulation; black, no regulation; black with white X, no value calculated.

For five selected samples, we checked the expression of the CLDN8 gene and several cytokine-encoding genes after the 0.5-h challenge (Fig. 6A through F). The most obvious difference was observed between samples ML 23 and ML 2. The latter elicited strong proinflammatory cytokine response by upregulating the genes encoding IL6, IL8, TNF-α, and IL1RN, whereas the ML 23 sample had no effect on the cells. Indeed, in cluster analysis, ML 23 clustered together with the control samples, whereas all ML 2 samples were clustered together with ML 9 and ML 15 (Fig. 6G). In should also be noted that ML 15 stood out with induction of mixed response in which besides upregulated pro-inflammatory IL6 and TNF-α, also anti-inflammatory IL10 and IL1RN were upregulated.

## DISCUSSION

A prerequisite for *in vitro* research of the MG microbial colonization and adherence is the availability of a cell culture model with well-characterized growth characteristics, a well-described epithelial surface with robust formation of the epithelial barrier which could sustain the addition of conditioned media or milk on the apical side of the cells. The cells should as much as possible resemble the *in vivo* situation from the surface point of view. Although several human mammary epithelial cell lines are currently available on the market or freely upon request, the properties of the MCF10A cell line grown on PCCM described so far (12–14, 18) seem promising and the best option for studying bacterial adhesion and biofilm formation. However, this cell line grown on PCCM has not yet been used for bacterial adhesion and biofilm formation studies, so the lack of adequate characterization of the epithelial surface, including mucin expression, needed to be addressed first.

We confirmed previous observations by A. Marshall et al. (12) which reported that MCF10A cells grown on PCCM form the tight junction barrier with high TEER and low FITC influx. In line with this was also the observation that the Claudin 8 gene was upregulated (eightfold) in comparison with confluent cells on 48-well CCP. Junctional claudins-3 and -8 are likely responsible for the very low paracellular permeability of the lactating MG, and the mRNA for Claudin-8 increased more than 25-fold, making it the most highly expressed claudin mRNA in the lactating mouse gland (19). In the present study, MCF10A cells reached their maximum TEER between days 14 and 17 post-seeding with an average plateau TEER between 1,200 and 2,300 Ω × cm^2^, which is lower than those reported in previous studies (12–14). This could be due to the different passages used for the experiments or differences in the chemicals used to prepare the cell culture media. In our study, we used the 8^th^–14^th^ passages after purchase (105^th–^111^th^ passages in total), whereas other studies did not specify the exact passage used.

The surface of the differentiated epithelial cells was morphologically diverse with at least two cell types and two types of microvilli. Similar observations were previously reported by A. Marshall et al. (12). Interestingly, epithelial cells obtained from isolated secretory alveoli from the bovine MG consisted of similar two types of epithelial cells (20). We also observed a third type of cells which were squeezed between the filter and the apical cell layer (Fig. 2C and D). The size and shape of these squeezed type of cells resembled stem-like/progenitor cells, which have also been described previously for this cell line (12, 21).

Analysis of the expression (Fig. 1) of mucins showed the upregulation of three mucin genes (MUC1, MUC4, and MUC20) as a result of growth on PCCM, indicating that the surface of these cells is covered with mucins. This was also confirmed by Alcian blue/PAS staining of membrane sections (Fig. S3) and SEM micrographs of samples that were not extensively washed (Fig. 2E and F). Expression of mucins in MCF10A cells is not well described in the literature, with the exception of MUC1. This cell line expresses endogenous MUC1, but to a lesser extent than carcinoma cells when cultured on CCP (22), but the expression is increased when cultured in Matrigel (21) or on PCCM (12). In fact, when MUC1 is considered as a marker for tumorigenesis, MCF10A cell culture is usually used as a negative control (23). Recent data on human breast tissues from 11 healthy women stored into the Human Protein Atlas database (24) showed that breast glandular cells express multiple mucins such as MUC1, MUC15, MUC4, MUC6, MUC5B, MUC16, and MUC20 above 10 normalized transcripts per million coding genes (nTPM) (25). This suggests that MCF10A grown on PCCM exhibit similar surface properties, to some extent resemble the normal physiological state of the MG, and thus may be suitable for studies on bacterial adhesion and biofilm development.

In the normal physiological state, mammary cells are in contact with excreted milk and present milk microbiota. Since A. Marshall et al. (12) reported that the composition of the upper chamber had little effect on maintaining membrane barrier function when only the medium in the lower chamber was changed daily, we speculated that MCF10A cells might withstand the challenge with HM, i.e., survive and retain barrier function. Indeed, we showed that the MAGIC model was able to withstand the challenge with heat-treated HM samples; however, there were noticeable differences among the effects of different milk samples, indicating that different bioactive components might be present in HM samples (26). As expected, when testing the raw pooled milk sample (Fig. 3A), bacterial overgrowth negatively affected on MCF10A cells, most probably due lactic acid production and the consequent drop of pH. Other bacterial metabolites and actions of pathogenic bacteria might also contribute to this effect. The negative effect was mitigated slightly with the addition of antibiotics. The initial bacterial concentration in the pooled milk sample (4.8 × 10^5^ CFU) was only slightly reduced after 24 h (6.2 × 10^4^ CFU) indicating that antibiotics only prevented the growth instead of killing of the bacteria. In contrast, heat-treated HM samples sustained the challenge for the first 3 h, but in later timepoints, noticeable differences were observed between different samples (pooled milk, ML3, and ML17), suggesting that non-bacterial factors and dead bacteria may also have negative impact on epithelial resistance of the MCF10A cells; however, it seems that the time needed for the expression of these effects might be longer (27).

Bacterial adhesion to mammary cells plays an important role in the establishment of the HM microbiota and in the generation of microbial dysbiosis which leads to pathological conditions such as mastitis (28). Therefore, we performed conventional adhesion experiments but used raw HM directly instead of pure bacterial cultures. We obtained pioneering data on which bacteria directly from HM microbiota are able to adhere to mammary epithelium *in vitro*. The major benefit of performing the experiments with whole breast milk samples is that the adhesion ability of bacteria is tested directly in their environment which includes various milk factors that are unique for each milk, such as human milk oligosaccharides, and also potential interbacterial interactions that might influence the adhesion ability on the mammary gland surface. The prevalence of culture-positive samples after challenging the MAGIC model with different HM samples was higher on BA than on TSA and WCA-M media (Fig. 4B), suggesting that the nutrients in BA or anaerobic conditions provide favorable conditions for the growth of these bacteria. The average adhesion ratio in culture-positive samples ranged from 0.22% (TSA) to 2.0% (WCA), which is within the range of other studies on adhesion to mammary epithelial cells (29, 30). It is important to note that the microbial profile of bacteria adhered to the surface of the MAGIC model reflected the microbiological profile of the input HM microbiota (Fig. 4A). In addition, the bacterial load in HM was positively correlated with the number of bacteria adhered on BA. All detected species can be found in HM or are common representatives of the skin, oral, respiratory, and gut microbiota (3, 31). Although *Staphylococcus*, *Streptococcus*, *Propionibacterium*, and *Corynebacterium* species are considered as part of the normal HM microbiota (3), there is still a question whether these bacteria adhere and permanently colonize the MG (32). From our experiment, we may conclude that some bacteria that are members of the normal HM microbiota are not well adapted to adhere to MG epithelium and are not permanent colonizers. For example, the adhesion rate of *S. epidermidis* was much higher compared with *Corynebacterium tuberculostearicum*. Microorganisms differ in their adhesion mechanisms and often show a preference for certain host glycan moieties according to the specific affinities of microbial glycan-binding proteins or lectins on the cell surface (33). The fact that *Corynebacterium* species differ greatly in their ability to adhere to different mucus glycans (33, 34) may provide an explanation for the lower adherence of *Corynebacterium*. SEM micrographs showed that not only live bacteria but also dead or damaged bacteria can adhere and interact with the epithelium (Fig. 5). Since the concept of postbiotics has received a considerable attention in recent years (35), it is important to recognize that dead probiotic bacteria may also be useful for improving the health of MG.

Since SEM micrographs clearly showed a direct interaction between bacterial cells and MCF10A epithelial cells, we analyzed the cellular response of the model after the challenge with raw HM. The expression of cytokines after a 0.5-h challenge reflected the differences in TEER. We observed three types of responses, low response in cytokine expression and no change in TEER with sample ML 23, a proinflammatory response and decrease in TEER with ML 2/ML 9, and a mixed response and no apparent change in TEER with ML 15. The resident milk microbiota is one of the most important drivers of cytokine response in MG (36). However, other bioactive compounds present in human milk may also contribute to the differences in cytokine expression. For example, various milk components such as vitamin D (37) or conjugated linoleic acid (38) can trigger an anti-inflammatory response with increased IL10 production. Although most studies of cytokine responses in the MG have been performed on bovine mammary epithelium *in vivo*, *ex vivo*, and *in vitro* (39), we can expect similar mechanisms. The hallmark of the mammary cytokine response to infection with *Escherichia coli* and its lipopolysaccharides is fast upregulation of expression of IL1-β, IL6, and TNF-α, whereas *Staphylococcus aureus* and lipoteichoic acid elicit a delayed response dominated by IL6 (40, 41).

The fact that HM microbiota of samples used in this study has already been characterized in detail (17) allowed us to hypothetically identify microbial factors that might drive the differences in cellular responses in our model. The microbiota composition of ML 23 was characterized with predominance of *Streptococcus* (16 rRNA gene next-generation sequencing (NGS); Fig. S4) and equilibrium of cultivable *Staphylococcus* and *Streptococcus* (Fig. S5). A very recent study reported that streptococci were associated with a higher IL10 in HM (36). Thus, the ML 23 microbiota might represent a “healthy,” balanced microbiota eliciting almost no cytokine response. In contrast, ML 2 was characterized by high aerobic bacterial load (Table S5), low diversity, and the predominance of cultivable environmental Gram-negative bacteria such as *Klebsiella* and *Ochrobactrum* (Fig. S4 and S5). These bacteria have also been reported as opportunistic pathogens (42) and causative agents of mastitis (43–46). In addition, the donor of the ML 2 milk sample complained of difficulties in expressing the milk, suggesting that the ML 2 microbiota represents an imbalanced microbiota with plausible mastitis endpoint. In the ML 15 sample, the microbiota diverged with the predominance of *Acinetobacter* (16 rRNA gene NGS; Fig. S4) and cultivable *Stenotrophomonas* (Fig. S5), which may explain the mixed cytokine response of the model. *Acinetobacter* is a normal inhabitant of the HM microbiota, and the healthy controls had more *Acinetobacter* in comparison to the mastitis group (45). However, *Stenotrophomonas* was found to be more abundant in the mastitis microbiota of humans (44) and cows (47). Overall, we can conclude that the MAGIC model provides an excellent starting point for further studies of the cytokine response of MG cells to individual biological components in HM, individually or as a whole.

It is important to point out the limitations of the MAGIC model. The most important feature of any *in vitro* model is to simulate as close as possible the natural situation. As highlighted in previous studies, MCF10A cells express luminal and basal markers, and therefore, this model may not be suitable for all applications (21). However, since these cells grown on PCCM gain several important characteristics of the *in vivo* environment (12), such as barrier formation, luminal vs. basal cell phenotypes, and expression of surface mucins, we believe that due to differentiation to a stage resembling ductal epithelium, they are highly suitable for microbial colonization studies. In addition, bacteria are expected to form biofilm in ducts (48, 49) rather than on the alveolar surface of the MG. It is also important to note that to assess the applicability of this model, we thoroughly tested only a small number of samples and checked for cytokine expression in a predefined time window (0.5 h). Several limitations of these results should be emphasized. In our experiment, bacteria that might pass inside the cell would be also considered as adherent; however, it is believed that the contribution of internalized bacteria to adherence is very low (50). Although our results suggest that bacteria might be the major driver of the observed effect on the MCF10A cells (Fig. 3C), we cannot clearly separate the effect of human milk microbiota and the effect of other milk components. In addition, physiological differences between the model and the mammary tissue may also influence the adhesion of bacteria from HM. Therefore, further verification of this model is needed to address all the issues raised in this study, such as what are the characteristics of the attached bacteria, whether these bacteria attach to MG *in vivo*, and how the cells respond in later time windows. Nonetheless, most of the literature on bacterial adherence to MG epithelial cells focuses on mastitis-causing bacterial strains, while the holistic approach on this topic has not yet been performed and opens many new research opportunities.

### Conclusions

In conclusion, the MAGIC model may be particularly useful for studies of bacterial attachment to the surface of the mammary ducts and for *in vitro* studies of biofilm formation and the development of the human MG microbiota. In addition, the differential cytokine response of the model after the challenge with raw HM suggests that the model is also useful for immunological studies of the interaction between bacteria and MG cells. We obtained pioneering information on which of the bacteria present in the raw HM were able to attach to the epithelium treated directly with raw HM, as well as on the effects of bacteria on the epithelial cells of the MAGIC model. The MAGIC cell model also offers new opportunities for research in other areas of MG physiology, such as the effects of bioactive milk components on microbial colonization of the MG, mastitis prevention, and studies of probiotic development. The model could be further upgraded with the addition of macrophages (51) in lower compartment for additional immunological studies. Since studies show that resident MG bacteria may be an important factor in breast cancer development, the MAGIC *in vitro* tool also offers new research opportunities in cancer research and thus reaching wider research applicability. For example, the model could be modified with breast cancer cell lines to explore the effect of microbiota on tumor outcomes.

## MATERIALS AND METHODS

### Cell culture on PCCM and sodium fluorescein flux measurements

An immortalized human mammary epithelial cell line, MCF10A, was purchased from ATTC (97th passage). Cells were grown as reported by Marshall et al. (12). Briefly, the normal complete growth medium (cF12) for MCF10A cells was DMEM:F12 (Sigma) with 2 mM stable glutamine dipeptide (Gibco), containing 5% horse serum (Gibco), insulin (10 µg/mL; Sigma), hydrocortisone (0.5 µg/mL; Sigma), epithelial growth factor (EGF; 20 ng/mL; Sigma), 100 IU/mL penicillin, and 0.1 mg/mL streptomycin (Sigma). Cells were grown in 25-cm^2^ cell culture flasks (VWR) in a monolayer to 90%–95% confluency, trypsinized, and reseeded into the next flask (one passage). Cells in 8^th^–15^th^ laboratory passage (105^th–^111^th^ cumulative) were counted for seeding onto PCCM (Transwell, Corning; 0.4-µm pores, polyester) or a 48-well plate in cF12. From growth optimization experiments, we concluded that the seeding concentration of MCF10A cells on PCCM in the range between 6.0 × 10^4^ and 1.3 × 10^5^ cells did not affect profoundly the development of TEER (Table S1). Media were changed in both chambers strictly on a 24-h schedule since changing the media every 2 days resulted in slower TEER development. During the optimization experiments, the cells were manipulated in laminar hood flow at room temperature or at 37°C by using a heating pad (Fig. S1A). Afterwards, all the manipulations were carried out on a heating pad at 37°C. TEER was measured daily after media change, from the 10th day on, with the Millicell-ERS Volt/Ohm meter (EMD Millipore).

For fluorescein flux experiment, cells were seeded onto PCCM in cF12. Every 2 days of culturing on PCCM, bottom side media were replaced with fresh cF12 medium supplemented with 0.1 mg/mL fluorescein sodium salt (NaFLU-cF12; Sigma) and cells were incubated at normal conditions. After 1 h, 100 µL of the medium on the apical side was transferred to a 96-well plate and analyzed with a fluorescence plate reader (excitation/emission 485 nm/530 nm; Tecan). The results were compared with those of the negative control, which was the PCCM chamber without cells and treated as experimental chambers (Fig. S1B).

### RNA extraction and gene expression analysis

#### RNA extraction and cDNA synthesis

In the cell growth experiment, after seeding the cells on PCCM (day 0), RNA was extracted every second day (days 1, 3…) for evaluation of gene expression. For comparison, RNA was extracted from confluent cells, grown on 48-well CCP. The experiment was performed in three independent experiments with two biological and two technical replicates.

In HM challenge experiment, the protocol for bacterial adhesion was followed except that the apical side of the cells was washed four times with 500 µL of HBSS with several in/out pipetting steps 30 minutes after HM addition.

In both experiments, the media or HM from both sides of the membrane were removed and 0.4 mL of TRIzol Reagent (Thermo Fisher) was added to the apical side of the PCCM. Cells were resuspended by pipetting, harvested, and stored at −70°C until further analysis. Total RNA was isolated according to the manufacturer’s instructions. RNA quantity and quality were evaluated with NanoVue (GE Healthcare). DNase treatment was performed according to the manufacturer’s instructions (Thermo Fisher). cDNA synthesis was carried out with a random mix of primers using the high-capacity cDNA reverse transcription kit with RNase inhibitor (Thermo Fisher).

#### Primer design

The sequences of RT-qPCR primers, used for mRNA quantification in this study, were obtained from PrimerBank (52), RTPrimerDB (53), and Primer-BLAST (54) and synthesized by IDT (Integrated DNA Technologies). Primer specificity and amplification efficiency were validated empirically with melting curve, agarose gel, and standard curve analysis of five serial dilutions from a pool of analyzed samples. Primer information is reported in Table S2.

#### RT-qPCR

RT-qPCR was preformed according to the minimum information for publication of quantitative real-time PCR experiments guidelines (55). RT-qPCR was performed in a 96-well format on a CFX96TM Real-Time System (Bio-Rad) using FastStart Universal SYBR Green Master (Merck). The PCR reaction consisted of 10 µL of FastStart Universal SYBR Green Master, 7 µL of PCR grade water, 0.5 µL of each primer (10 µM), and 2 µL of cDNA (20× diluted in PCR grade water) in a total volume of 20 µL. In the initial screening for expression of mucin genes, the pooled DNA sample from each timepoint was used as a template. Only mucin genes with low Ct were analyzed for differential gene expression (Table S3). All samples for the differential gene expression analysis were assayed in duplicates, and a no-template control was included with each run. Cycling conditions were as follows: 10 minutes at 95°C followed by 40 rounds of 15 s at 95°C, 30 s at 60°C, and 30 s at 72°C. Melting curve analysis for determining the dissociation of PCR products was performed from 65°C to 95°C. All samples with large deviations within technical replicates were excluded from further analysis. The normalized relative gene expression (ΔΔCq) was calculated with Bio-Rad CFX Maestro 1.1 according to M. W. Pfaffl (56). The geometric mean of three internal reference genes (HPRT1, GAPDH, and ACTB) was used for normalization (57).

The results are presented as fold of normalized expression relative to cells confluent on 48-well CCP or in the case of HM challenge experiment, to cells with no HM addition. Statistical comparison between samples from different timepoints and CCP was performed with GraphPad Prism 5 (GraphPad Software) by using non-parametric one-way ANOVA (Kruskal-Wallis statistic) and Dunn’s multiple comparison test with adjustments of *P*-values to account for multiple comparisons. A *P*-value of <0.05 was considered statistically significant. Change in gene expression higher that twofold was considered as biologically relevant.

### Scanning electron microscopy

Cells were grown on PCCM until TEER reached a plateau. In the adhesion experiment, cells were challenged with a raw HM sample. Cells on the membranes were washed (if indicated) two times with HBSS and three times (for 1 minute) with sodium cacodylate buffer (0.1 M, pH 7.4). Afterwards, we fixed the cells with 1.0% (vol/vol) glutaraldehyde and 0.4% (vol/vol) formaldehyde in 0.1 M sodium phosphate buffer (pH 7.2) at 4°C overnight. After washing in the buffer, the samples were post-fixed in 1% aqueous solution of OsO_4_ (SPI-CHEM). After three washings in demineralized water, post-fixed samples were dehydrated in an ethanol series of ascending concentrations (50%, 70%, 80%, 90%, and 96%). The final ethanol concentration was gradually replaced by hexamethyldisilazane (Merck) and allowed to air dry overnight. PCCM were cut out and attached to the metal holders with silver paint (SPI CHEM), coated with platinum on a SCD 050 sputter coater (BAL-TEC), and observed with a JEOL JSM-7500F (JEOL Ltd.) field emission scanning microscope.

### Challenge of differentiated MCF10A with raw and sterilized HM samples

HM samples were collected in the previous study (17) in which HM microbiota was determined by 16S rRNA gene NGS and cultivation/MALDI-TOF mass spectrometry identification. The study was conducted according to the guidelines of the Declaration of Helsinki. All of the procedures involving human subjects were approved by the National Medical Ethics Committee of the Republic of Slovenia (0120-328/2017/3). Briefly, we used 16 HM samples from healthy mothers from the central Slovenia region who donated one HM sample (at least 25 mL), between the 3^rd^ and 8^th^ week of lactation. After signing the informed consent, mothers were asked to collect the HM sample from both breasts, either by manual expression or with a breast pump (not provided). They were instructed to clean each breast with warm water and soap, discard the first drop before collecting the sample, and freeze the sample immediately after collection. At the same time, mothers were asked to complete a short questionnaire. Samples were transported to the laboratory within 1 week after collection and frozen at −70°C until further analysis.

The pooled HM sample was prepared by pooling 1 mL of each raw HM sample. Heat treatment of samples was performed in autoclave at 110°C, for 15 minutes. The pooled HM sample with the addition of antibiotics was prepared immediately before the experiment by mixing antibiotics [100 IU/mL of penicillin, 0.1 mg/mL of streptomycin, 0.1 mg/mL of kanamycin (Sigma), and 10 µg/mL of tetracycline (Sigma)] with pooled HM in a thermal shaker (37°C, 600 × rpm, 10 min). The experiment was performed after TEER was stable and reached more than 1,000 Ω × cm^2^. One day before the experiment, the cF12 was changed with cF12 without antibiotics (cF12-ATB). On the day of the experiment, cF12-ATB was changed once more and TEER was measured. Media on the apical side of the MCF10A cells were replaced with 500 µL of the treated HM sample or by cF12 with added kanamycin and tetracycline or by cF12-ATB (control). TEER was measured on different timepoints for up to 24 h. We tested the microbial load in raw HM, after the heat or antibiotic treatment and before and at the end of the experiment by plating on TSA (Biolife Italiana).

### Adhesion of bacteria from HM microbiota and identification of adhered bacteria

The experiment was performed in the same manner as the challenge experiment. HM was thawed in a thermostatic shaker (37°C, 600 × rpm, 6 min), and media on the apical side of the MCF10A cells were replaced with 500 µL of milk. TEER was measured immediately after the replacement and after 1 h of incubation on 37°C/5% CO_2_. In pre-experiments, we determined the optimal timepoint for the adhesion test in several pre-experiments (Table S4). The substantial bacterial growth in raw HM at 37°C started between 1.5 and 2 h. To prevent misinterpretation of adherence due to bacterial growth, we selected the 1-h timepoint to test bacterial adhesion. After 1 h, HM on the apical side and medium on the basolateral side were sucked out with a vacuum pump. The apical side of the cells was washed 4× with 500 µL of HBSS with several in/out pipetting steps (1^st^ time 3×, 2^nd^ time 2×, 3^rd^ time 1×, 4^th^ time 1× upper side with 500 µL, and lower side 1 mL of HBSS). Cells were trypsinized by adding 300 µL of Trypsin (0.025% solution in HBSS) on both sides of the PCCM. After 20 minutes, cells were homogenized with pipetting, and after 30 minutes, trypsin from the basolateral side was sucked out, and 200 µL of Triton X-100 (0.05% + 5% horse serum) was added to the apical side of the cells for 15 minutes. Finally, the suspension of cells (500 µL) was put immediately on ice and plated on BA [Columbia agar + 5% sheep blood (BioMérieux, Marcy l’Etoile, France)], TSA, WCA-M (Oxoid, Basingstoke, UK) supplemented with mupirocin (50 mg/L) (AppliChem), and ROG agar (pH 5.5; Merck). Plates were incubated at 37°C for 72 h in aerobic (TSA) conditions or anaerobic (BA, WCA-M, and ROG) conditions. Colonies with various morphology were systematically picked up from TSA (15 colonies), BA (15 colonies), WCA, and ROG (up to 6 colonies) and analyzed by the matrix-assisted laser desorption/ionization time-of-flight mass spectrometry (MALDI-TOF MS) (Microflex LT system; Bruker Daltonics) according to the manufacturer’s instructions and previously described (17). Each sample was tested on four PCCM. The growth of bacteria in HM during 1-h incubation was tested for each sample, i.e., on PCCM without cells (Table S5). Each plate also had negative control for TEER measurements, i.e., confluent cells without treatment.

## Data Availability

All data generated or analyzed during this study are included in this published article (and its supplementary information files).

## SUPPLEMENTAL MATERIAL

Supplementary tables and figures spectrum.02369-23-s0001.pdf Supplementary Figures S1-S5 and Supplementary Tables S1-S7.

ASM does not own the copyrights to Supplemental Material that may be linked to, or accessed through, an article. The authors have granted ASM a non-exclusive, world-wide license to publish the Supplemental Material files. Please contact the corresponding author directly for reuse.

## References

[1] Consales A, Cerasani J, Sorrentino G, Morniroli D, Colombo L, Mosca F, Giannì ML. 2022. The hidden universe of human milk microbiome: origin, composition, determinants, role, and future perspectives. Eur J Pediatr 181:1811–1820. doi:10.1007/s00431-022-04383-1 35124754 PMC9056486

[2] Edwards CA, Van Loo-Bouwman CA, Van Diepen JA, Schoemaker MH, Ozanne SE, Venema K, Stanton C, Marinello V, Rueda R, Flourakis M, Gil A, Van der Beek EM. 2022. A systematic review of breast milk microbiota composition and the evidence for transfer to and colonisation of the infant gut. Benef Microbes 13:365–381. doi:10.3920/BM2021.0098 36377578

[3] Fernández L, Pannaraj PS, Rautava S, Rodríguez JM. 2020. The microbiota of the human mammary ecosystem. Front Cell Infect Microbiol 10:586667. doi:10.3389/fcimb.2020.586667 33330129 PMC7718026

[4] Kerro Dego O, Pacha PA, Gillespie BE, Pighetti GM. 2020. Experimental Staphylococcus aureus mastitis infection model by teat dipping in bacterial culture suspension in dairy cows. Animals (Basel) 10:751. doi:10.3390/ani10050751 32344845 PMC7277341

[5] Notebaert S, Meyer E. 2006. Mouse models to study the pathogenesis and control of bovine mastitis. A review. Vet Q 28:2–13. doi:10.1080/01652176.2006.9695201 16605156

[6] Huynh HT, Robitaille G, Turner JD. 1991. Establishment of bovine mammary epithelial cells (MAC-T): an in vitro model for bovine lactation. Exp Cell Res 197:191–199. doi:10.1016/0014-4827(91)90422-q 1659986

[7] de Almeida MHR, Bortolotto GDS, Dutra RC, Guimarães GN, Felipetti FA. 2021. Cell culture of the normal human mammary gland cultivated in monolayer - a mini systematic review. Acta Histochem 123:151798. doi:10.1016/j.acthis.2021.151798 34666236

[8] Betts CB, Pennock ND, Caruso BP, Ruffell B, Borges VF, Schedin P. 2018. Mucosal immunity in the female murine mammary gland. J Immunol 201:734–746. doi:10.4049/jimmunol.1800023 29884705 PMC6036228

[9] Wagner CE, Wheeler KM, Ribbeck K. 2018. Mucins and their role in shaping the functions of mucus barriers. Annu Rev Cell Dev Biol 34:189–215. doi:10.1146/annurev-cellbio-100617-062818 30296390 PMC11906035

[10] Puleo J, Polyak K. 2021. The MCF10 model of breast tumor progression. Cancer Res 81:4183–4185. doi:10.1158/0008-5472.CAN-21-1939 34400468

[11] Soule HD, Maloney TM, Wolman SR, Peterson WD, Brenz R, McGrath CM, Russo J, Pauley RJ, Jones RF, Brooks SC. 1990. Isolation and characterization of a spontaneously immortalized human breast epithelial cell line, MCF-10. Cancer Res 50:6075–6086. 1975513

[12] Marshall AM, Pai VP, Sartor MA, Horseman ND. 2009. In vitro multipotent differentiation and barrier function of a human mammary epithelium. Cell Tissue Res 335:383–395. doi:10.1007/s00441-008-0719-0 19005683

[13] Pai VP, Horseman ND. 2008. Biphasic regulation of mammary epithelial resistance by serotonin through activation of multiple pathways. J Biol Chem 283:30901–30910. doi:10.1074/jbc.M802476200 18782769 PMC2576527

[14] Stull MA, Pai V, Vomachka AJ, Marshall AM, Jacob GA, Horseman ND. 2007. Mammary gland homeostasis employs serotonergic regulation of epithelial tight junctions. Proc Natl Acad Sci U S A 104:16708–16713. doi:10.1073/pnas.0708136104 17940054 PMC2034263

[15] Chung HH, Mireles M, Kwarta BJ, Gaborski TR. 2018. Use of porous membranes in tissue barrier and co-culture models. Lab Chip 18:1671–1689. doi:10.1039/c7lc01248a 29845145 PMC5997570

[16] Langerholc T, Maragkoudakis PA, Wollgast J, Gradisnik L, Cencic A. 2011. Novel and established intestinal cell line models - an indispensable tool in food science and nutrition. Trends Food Sci Technol 22:S11–S20. doi:10.1016/j.tifs.2011.03.010 32336880 PMC7172287

[17] Treven P, Mahnič A, Rupnik M, Golob M, Pirš T, Matijašić BB, Lorbeg PM. 2019. Evaluation of human milk microbiota by 16S rRNA gene next-generation sequencing (NGS) and cultivation/MALDI-TOF mass spectrometry identification. Front Microbiol 10:2612. doi:10.3389/fmicb.2019.02612 31803156 PMC6872673

[18] Chin AR, Yan W, Cao M, Liu X, Wang SE. 2018. Polarized secretion of extracellular vesicles by mammary epithelia. J Mammary Gland Biol Neoplasia 23:165–176. doi:10.1007/s10911-018-9402-6 29968174 PMC6103817

[19] Baumgartner HK, Rudolph MC, Ramanathan P, Burns V, Webb P, Bitler BG, Stein T, Kobayashi K, Neville MC. 2017. Developmental expression of claudins in the mammary gland. J Mammary Gland Biol Neoplasia 22:141–157. doi:10.1007/s10911-017-9379-6 28455726 PMC5488167

[20] Ditcham WG, Leigh JA, Bland AP, Hill AW. 1996. Adhesion of Streptococcus uberis to monolayers of cultured epithelial cells derived from the bovine mammary gland. FEMS Immunol Med Microbiol 14:145–150. doi:10.1111/j.1574-695X.1996.tb00281.x 8809550

[21] Qu Y, Han B, Yu Y, Yao W, Bose S, Karlan BY, Giuliano AE, Cui X. 2015. Evaluation of MCF10A as a reliable model for normal human mammary epithelial cells. PLoS One 10:e0131285. doi:10.1371/journal.pone.0131285 26147507 PMC4493126

[22] Ahmad R, Raina D, Trivedi V, Ren J, Rajabi H, Kharbanda S, Kufe D. 2007. MUC1 oncoprotein activates the IkappaB kinase beta complex and constitutive NF-kappaB signalling. Nat Cell Biol 9:1419–1427. doi:10.1038/ncb1661 18037881 PMC4209910

[23] Zhang Y, Guo S, Huang H, Mao G, Ji X, He Z. 2018. Silicon nanodot-based aptasensor for fluorescence turn-on detection of mucin 1 and targeted cancer cell imaging. Anal Chim Acta 1035:154–160. doi:10.1016/j.aca.2018.06.032 30224134

[24] Karlsson M, Zhang C, Méar L, Zhong W, Digre A, Katona B, Sjöstedt E, Butler L, Odeberg J, Dusart P, Edfors F, Oksvold P, von Feilitzen K, Zwahlen M, Arif M, Altay O, Li X, Ozcan M, Mardinoglu A, Fagerberg L, Mulder J, Luo Y, Ponten F, Uhlén M, Lindskog C. 2021. A single-cell type transcriptomics map of human tissues. Sci Adv 7:eabh2169. doi:10.1126/sciadv.abh2169 34321199 PMC8318366

[25] Bhat-Nakshatri P, Gao H, Sheng L, McGuire PC, Xuei X, Wan J, Liu Y, Althouse SK, Colter A, Sandusky G, Storniolo AM, Nakshatri H. 2021. A single-cell atlas of the healthy breast tissues reveals clinically relevant clusters of breast epithelial cells. Cell Rep Med 2:100219. doi:10.1016/j.xcrm.2021.100219 33763657 PMC7974552

[26] Oberčkal J, Liaqat H, Matijašić BB, Rozman V, Treven P. 2023. Quantification of lactoferrin in human milk using monolithic cation exchange HPLC. J Chromatogr B Analyt Technol Biomed Life Sci 1214:123548. doi:10.1016/j.jchromb.2022.123548 36476358

[27] Ramage G, Lappin DF, Millhouse E, Malcolm J, Jose A, Yang J, Bradshaw DJ, Pratten JR, Culshaw S. 2017. The epithelial cell response to health and disease associated oral biofilm models. J Periodontal Res 52:325–333. doi:10.1111/jre.12395 27330034 PMC5412879

[28] Pedersen RR, Krömker V, Bjarnsholt T, Dahl-Pedersen K, Buhl R, Jørgensen E. 2021. Biofilm research in bovine mastitis. Front Vet Sci 8:656810. doi:10.3389/fvets.2021.656810 34026893 PMC8138050

[29] Fessia AS, Odierno LM. 2022. Evaluation of the relative expression of genes associated with adherence after different hours of co-culture between Streptococcus uberis and MAC-T cells. Microbes Infect 24:104914. doi:10.1016/j.micinf.2021.104914 34864211

[30] Queiroga MC, Duarte EL, Laranjo M. 2018. Sheep mastitis Staphylococcus epidermidis biofilm effects on cell adhesion and inflammatory changes. Small Rumin Res 168:6–11. doi:10.1016/j.smallrumres.2018.09.009

[31] Whitman WB, Vos P, Dedysh S, Hedlund B, Kämpfer P, Rainey F, Trujillo ME, Bowman JP, Brown DR, Glöckner FO, Oren A, Paster BJ, Wade W, Ward N, Busse H-J, Reysenbach A-L. 2015. Bergey's manual of systematics of archaea and bacteria. John Wiley & Sons, Hoboken, New Jersey.

[32] Moossavi S, Azad MB. 2020. Origins of human milk microbiota: new evidence and arising questions. Gut Microbes 12:1667722. doi:10.1080/19490976.2019.1667722 31684806 PMC7524145

[33] Lin C-H, Peterson RA, Gueniche A, de Beaumais SA, Hourblin V, Breton L, Dalko M, Packer NH. 2019. Differential involvement of glycans in the binding of Staphylococcus epidermidis and Corynebacterium spp. to human sweat. Microbiol Res 220:53–60. doi:10.1016/j.micres.2018.12.007 30744819

[34] Salamzade R, Swaney MH, Kalan LR. 2023. Comparative genomic and metagenomic investigations of the Corynebacterium tuberculostearicum species complex reveals potential mechanisms underlying associations to skin health and disease. Microbiol Spectr 11:e0357822. doi:10.1128/spectrum.03578-22 36541755 PMC9927478

[35] Salminen S, Collado MC, Endo A, Hill C, Lebeer S, Quigley EMM, Sanders ME, Shamir R, Swann JR, Szajewska H, Vinderola G. 2021. The international scientific association of probiotics and prebiotics (ISAPP) consensus statement on the definition and scope of postbiotics. Nat Rev Gastroenterol Hepatol 18:649–667. doi:10.1038/s41575-021-00440-6 33948025 PMC8387231

[36] Cortés-Macías E, Selma-Royo M, Rio-Aige K, Bäuerl C, Rodríguez-Lagunas MJ, Martínez-Costa C, Pérez-Cano FJ, Collado MC. 2023. Distinct breast milk microbiota, cytokine, and adipokine profiles are associated with infant growth at 12 months: an in vitro host-microbe interaction mechanistic approach. Food Funct 14:148–159. doi:10.1039/d2fo02060b 36472137

[37] Alva-Murillo N, Téllez-Pérez AD, Medina-Estrada I, Alvarez-Aguilar C, Ochoa-Zarzosa A, López-Meza JE. 2014. Modulation of the inflammatory response of bovine mammary epithelial cells by cholecalciferol (vitamin D) during Staphylococcus aureus internalization. Microb Pathog 77:24–30. doi:10.1016/j.micpath.2014.10.006 25457796

[38] Ma N, Chang G, Huang J, Wang Y, Gao Q, Cheng X, Liu J, Shen X. 2019. cis-9, trans-11-conjugated linoleic acid exerts an anti-inflammatory effect in bovine mammary epithelial cells after Escherichia coli stimulation through NF-κB signaling pathway. J Agric Food Chem 67:193–200. doi:10.1021/acs.jafc.8b05500 30562023

[39] Rainard P, Gilbert FB, Germon P. 2022. Immune defenses of the mammary gland epithelium of dairy ruminants. Front Immunol 13:1031785. doi:10.3389/fimmu.2022.1031785 36341445 PMC9634088

[40] Günther J, Esch K, Poschadel N, Petzl W, Zerbe H, Mitterhuemer S, Blum H, Seyfert H-M. 2011. Comparative kinetics of Escherichia coli- and Staphylococcus aureus-specific activation of key immune pathways in mammary epithelial cells demonstrates that S. aureus elicits a delayed response dominated by interleukin-6 (IL-6) but not by IL-1A or tumor necrosis factor alpha. Infect Immun 79:695–707. doi:10.1128/IAI.01071-10 21115717 PMC3028868

[41] Reisinger N, Wendner D, Schauerhuber N, Mayer E. 2021. Effect of lipopolysaccharides (LPS) and lipoteichoic acid (LTA) on the inflammatory response in rumen epithelial cells (REC) and the impact of LPS on claw explants. Animals (Basel) 11:2058. doi:10.3390/ani11072058 34359186 PMC8300308

[42] Ryan MP, Pembroke JT. 2020. The genus Ochrobactrum as major opportunistic pathogens. Microorganisms 8:1797. doi:10.3390/microorganisms8111797 33207839 PMC7696743

[43] Ong SS, Xu J, Sim CK, Khng AJ, Ho PJ, Kwan PKW, Ravikrishnan A, Tan K-T, Tan QT, Tan EY, Tan S-M, Putti TC, Lim SH, Tang ELS, Nagarajan N, Karnani N, Li J, Hartman M. 2023. Profiling microbial communities in idiopathic granulomatous mastitis. Int J Mol Sci 24:1042. doi:10.3390/ijms24021042 36674562 PMC9863225

[44] Patel SH, Vaidya YH, Joshi CG, Kunjadia AP. 2016. Culture-dependent assessment of bacterial diversity from human milk with lactational mastitis. Comp Clin Pathol 25:437–443. doi:10.1007/s00580-015-2205-x

[45] Patel SH, Vaidya YH, Patel RJ, Pandit RJ, Joshi CG, Kunjadiya AP. 2017. Culture independent assessment of human milk microbial community in lactational mastitis. Sci Rep 7:7804. doi:10.1038/s41598-017-08451-7 28798374 PMC5552812

[46] Song J, Xiang W, Wang Q, Yin J, Tian T, Yang Q, Zhang M, Ge G, Li J, Diao N, Liu F, Shi K, Cai R, Du R, Gong Q. 2023. Prevalence and risk factors of Klebsiella spp. in milk samples from dairy cows with mastitis-a global systematic review. Front Vet Sci 10:1143257. doi:10.3389/fvets.2023.1143257 37035815 PMC10073557

[47] Kuehn JS, Gorden PJ, Munro D, Rong R, Dong Q, Plummer PJ, Wang C, Phillips GJ. 2013. Bacterial community profiling of milk samples as a means to understand culture-negative bovine clinical mastitis. PLoS One 8:e61959. doi:10.1371/journal.pone.0061959 23634219 PMC3636265

[48] Boix-Amorós A, Hernández-Aguilar MT, Artacho A, Collado MC, Mira A. 2020. Human milk microbiota in sub-acute lactational mastitis induces inflammation and undergoes changes in composition, diversity and load. Sci Rep 10:18521. doi:10.1038/s41598-020-74719-0 33116172 PMC7595153

[49] Rodríguez JM. 2014. The origin of human milk bacteria: is there a bacterial entero-mammary pathway during late pregnancy and lactation? Adv Nutr 5:779–784. doi:10.3945/an.114.007229 25398740 PMC4224214

[50] Tribelli PM, Luqman A, Nguyen M-T, Madlung J, Fan S-H, Macek B, Sass P, Bitschar K, Schittek B, Kretschmer D, Götz F. 2020. Staphylococcus aureus Lpl protein triggers human host cell invasion via activation of Hsp90 receptor. Cell Microbiol 22:e13111. doi:10.1111/cmi.13111 31515903

[51] Hassel C, Gausserès B, Guzylack-Piriou L, Foucras G. 2021. Ductal macrophages predominate in the immune landscape of the lactating mammary gland. Front Immunol 12:754661. doi:10.3389/fimmu.2021.754661 34745127 PMC8564477

[52] Spandidos A, Wang X, Wang H, Seed B. 2010. PrimerBank: a resource of human and mouse PCR primer pairs for gene expression detection and quantification. Nucleic Acids Res 38:D792–D799. doi:10.1093/nar/gkp1005 19906719 PMC2808898

[53] Lefever S, Vandesompele J, Speleman F, Pattyn F. 2009. RTPrimerDB: the portal for real-time PCR primers and probes. Nucleic Acids Res 37:D942–D945. doi:10.1093/nar/gkn777 18948285 PMC2686610

[54] Ye J, Coulouris G, Zaretskaya I, Cutcutache I, Rozen S, Madden TL. 2012. Primer-BLAST: a tool to design target-specific primers for polymerase chain reaction. BMC Bioinformatics 13:134. doi:10.1186/1471-2105-13-134 22708584 PMC3412702

[55] Bustin SA, Benes V, Garson JA, Hellemans J, Huggett J, Kubista M, Mueller R, Nolan T, Pfaffl MW, Shipley GL, Vandesompele J, Wittwer CT. 2009. The MIQE guidelines: minimum information for publication of quantitative real-time PCR experiments. Clin Chem 55:611–622. doi:10.1373/clinchem.2008.112797 19246619

[56] Pfaffl MW. 2001. A new mathematical model for relative quantification in real-time RT-PCR. Nucleic Acids Res 29:e45. doi:10.1093/nar/29.9.e45 11328886 PMC55695

[57] Vandesompele J, De Preter K, Pattyn F, Poppe B, Van Roy N, De Paepe A, Speleman F. 2002. Accurate normalization of real-time quantitative RT-PCR data by geometric averaging of multiple internal control genes. Genome Biol 3:research0034.1. doi:10.1186/gb-2002-3-7-research0034 12184808 PMC126239

