# Supplementary material for: In vitro model of human mammary gland microbial colonization (MAGIC) demonstrates distinctive cytokine response to imbalanced human milk microbiota
Source: Microbiol Spectr. 2024 Jan 30;12(3):e02369-23. doi: 10.1128/spectrum.02369-23 (PMC10913382; doi:10.1128/spectrum.02369-23)
Supplement: Supplementary tables and figures — Supplementary Figures S1-S5 and Supplementary Tables S1-S7. [file spectrum.02369-23-s0001.pdf]

In vitro model of human MAmmary Gland mIcrobial Colonization (MAGIC) demonstrates distinctive cytokine response to imbalanced human milk microbiota

Primož Treven*, Petra Mohar Lorbeg, Diana Paveljšek, Rok Kostanjšek; Majda Golob, Bojana Bogovič Matijašič

List of Supplementary file 1:

List of supplementary tables:

Table S1:	Spearman rank order correlations between number of cells seeded on TransWells and TEER development parameters
Table S2:	Primers used in this study
Table S3:	Initial screening for expression of mucin genes in 20 × diluted pooled cDNA sample.
Table S4:	Pre-experiment on growth of bacteria in the human milk sample ML 10 at 37 °C and their adhesion to differentiated MCF10A
Table S5:	Number of bacteria (colony forming units (CFU)), grown on BA, TSA, WCA-M and Rogosa agar in human milk samples and in culture-positive raw human milk samples and in trypsinised MCF10A cells after the 1-h adhesion assay.
Table S6:	Prevalence of genus-positive samples of raw human milk and of trypsinised MCF10A cells after the adhesion assay.
Table S7:	Prevalence of species-positive samples of raw human milk and of trypsinised MCF10A cells after the adhesion assay.

Supplementary table S1: Spearman rank order correlations between number of cells seeded on TransWells and TEER development parameters

	TEER at day 14	Day at TEER 1000	Day at peak TEER	Max TEER
**N (12-well TW plates)**	31	31	31	31
**Correlation Coefficient**	-0.3	0.228	0.275	-0.0428
**p-value**	0.1	0.216	0.134	0.817

Supplementary table S2: Primers used in this study

Gene	5'- 3'	Primer sequence	Product length (bp)	Primer efficiency [%][a]
HPRT1	fw	CCTGGCGTCGTGATTAGTGAT	131	98.8
	rw	AGACGTTCAGTCCTGTCCATAA		
ACTB	fw	GTCATTCCAAATATGAGATGCGT	121	98.8
	rw	GCTATCACCTCCCCTGTGTG		
GAPDH	fw	TGCACCACCAACTGCTTAGC	87	99.6
	rw	GGCATGGACTGTGGTCATGAG		
CDH1	fw	ATTTTTCCCTCGACACCCGAT	109	99.2
	rw	TCCCAGGCGTAGACCAAGA		
OCLN	fw	ACAAGCGGTTTTATCCAGAGTC	89	99.0
	rw	GTCATCCACAGGCGAAGTTAAT		
CLDN1	fw	CCTCCTGGGAGTGATAGCAAT	145	98.5
	rw	GGCAACTAAAATAGCCAGACCT		
CLDN8	fw	CAACCCATGCCTTAGAAATCGC	162	99.3
	rw	TCACGCAATTCATCCACAGTC		
MUC1	fw	TGCCGCCGAAAGAACTACG	76	98.6
	rw	TGGGGTACTCGCTCATAGGAT		
MUC2	fw	GAGGGCAGAACCCGAAACC	117	/
	rw	GGCGAAGTTGTAGTCGCAGAG		
MUC3A	fw	CTGGGATGAGGAAGTCGTGG	154	/
	rw	TCACACTGAGGACGAGGTCA		
MUC4	fw	CAGGCCACCAACTTCATCG	101	98.7
	rw	ACACGGATTGCGTCGTGAG		
MUC5AC	fw	AGCCGGGAACCTACTACTCG	165	/
	rw	AAGTGGTCATAGGCTTCGTGC		
MUC5B	fw	GCCCACATCTCCACCTATGAT	141	/
	rw	GCAGTTCTCGTTGTCCGTCA		
MUC6	fw	CTGCCCTATACCAGCAATGGA	147	/
	rw	CTGACCCATGTACTTCCGCTC		
MUC7	fw	CACCAGAAGCCGTTCATTAGAA	149	/
	rw	GGGTTGACCACACTGCTATTT		
MUC8	fw	GTTACCGGCTCACTCCTTCC	163	/
	rw	GAACAAACGCCTGGGTTGAG		
MUC12	fw	CTCGTGTATGGGATCGTGGG	109	/
	rw	CATACTGTTCCCTGTGCCGT		
MUC13	fw	ATGCGTGCTGATGACAAGTTT	153	/
	rw	ACACCGAAGGGTCAAATCATAGT		
MUC15	fw	TATTCACTTCTATCGGGGAGCC	175	/
	rw	GGGAATGACTCGCCTTGAGAT		
MUC16	fw	CCAGTCCTACATCTTCGGTTGT	161	101.5
	rw	AGGGTAGTTCCTAGAGGGAGTT		
MUC17	fw	TCTCAGCACGTTAGGACAGGT	115	/
	rw	TCGAGGTCATCTCAGGGTTGG		
MUC20	fw	ATCTCCCAACTTCATGGTGCT	104	100.3
	rw	TGCCTGTGATGGTCTGAACTG		
TNF-α	fw	GGCGTGGAGCTGAGAGATAAC	120	100.0
	rv	GGTGTGGGTGAGGAGCACAT		
IL6	fw	GGCACTGGCAGAAAACAACC	85	99.8
	rv	GCAAGTCTCCTCATTGAATCC		
IL8	fw	GAACTGAGAGTGATTGAGAGTGGA	134	98.3
	rv	CTCTTCAAAAACTTCTCCACAACC		
IL10	fw	GACTTTAAGGGTTACCTGGGTTG	112	102.5
	rv	TCACATGCGCCTTGATGTCTG		
IL1RN	fw	TTCCTGTTCCATTCAGAGACGAT	151	102.6
	rv	AATTGACATTTGGTCCTTGCAA		

[a]/ - not calculated due to high or no Ct in pooled sample

Supplementary table S3: Initial screening for expression of mucin genes in 20 × diluted pooled cDNA sample.

Only mucin genes with Ct lower than 30 were analyzed for differential gene expression.

Mucin	mean Cq	Cq st dev	Status
MUC1	21.34	0.004	analysed for expression
MUC4	30.06	0.069	analysed for expression
MUC16	24.67	0.049	analysed for expression
MUC20	29.59	0.197	analysed for expression
MUC3A	33.19	0.589	low signal
MUC5B	34.5	0.409	low signal
MUC2	36.47	1.433	low signal
MUC13	37.4	0.345	low signal
MUC5AC	no ct	no ct	no ct
MUC6	no ct	no ct	no ct
MUC7	no ct	no ct	no ct
MUC8	no ct	no ct	no ct
MUC12	no ct	no ct	no ct
MUC15	no ct	no ct	no ct
MUC17	no ct	no ct	no ct

Supplementary table S4: **Pre-experiment on growth of bacteria in the human milk sample ML 10 at 37 °C and their adhesion to differentiated MCF10A**

Media	Treatment	0,5 h	1 h	1,5 h	2h	3	4
TSA	24-well plate				1.20E+06	3.80E+06	1.90E+07
	filter with media on basolateral side			1.00E+06			2.60E+07
	milk on cells	1.00E+03	7.10E+03	2.10E+06			3.70E+07
	adhered bacteria	0.00E+00	0.00E+00	3.50E+03			3.40E+02
BA	milk on cells	2.00E+03	1.40E+05				
	adhered bacteria	1.00E+00	1.00E+01	5.90E+03			

Table S5: Number of bacteria (colony forming units (CFU)), grown on BA, TSA, WCA-M and Rogosa agar in human milk samples and in culture-positive raw human milk samples and in trypsinised MCF10A cells after the 1-h adhesion assay.

Samples were plated on Blood agar (BA), Tryptic Soy Agar (TSA), Wilkins-Chalgrene Anaerobe agar supplemented with mupirocin (WCA-M), and Rogosa (ROG) agar. Plates were incubated at 37°C for 72 h in aerobic (TSA) conditions or anaerobic conditions (BA, WCA-M and ROG).

Control milk - The growth of bacteria in milk during 1 h incubation.

Sample name	Quantity [CFU eq]*	TSA [CFU]	BA [CFU]	WCA-M [CFU]	Rog [CFU]	TSA [CFU]	BA [CFU]	WCA-M [CFU]	Rog [CFU]	Control TSA	Control BA
	Raw milk					Adhered bacteria				Control milk	
1	3.5E+05	3.3E+04	4.4E+04	6.50E+02	1.0E+01	2.8E+02	7.5E+01	1.5E+01	<10	3.9E+04	3.50E+04
2	3.2E+07	1.1E+07	1.2E+06	2.50E+04	1.0E+01	8.4E+03	6.3E+03	1.5E+03	<10	1.5E+07	1.40E+06
9	7.5E+05	6.3E+03	1.4E+05	3.70E+04	5.7E+02	<10	4.5E+01	1.3E+01	<10	7.1E+03	7.30E+04
10	1.6E+05	1.1E+04	9.4E+03	9.20E+02	<10	0.0E+00	1.0E+01	<10	<10	7.1E+03	1.40E+05
12	7.5E+04	9.5E+03	8.4E+03	8.00E+01	<10	0.0E+00	3.8E+01	<11	<10	4.7E+03	1.50E+04
13	1.5E+05	5.3E+04	4.4E+04	1.10E+03	2.0E+01	2.0E+01	1.4E+02	2.0E+01	<10	2.2E+04	5.40E+04
15	3.3E+06	9.1E+05	2.4E+05	1.40E+04	<10	1.1E+03	2.5E+02	<10	<10	8.5E+05	2.90E+05
16	6.8E+04	9.2E+04	8.1E+04	4.30E+04	<10	1.0E+01	1.2E+02	8.0E+01	<10	4.5E+03	6.50E+04
17	1.2E+05	1.1E+04	1.9E+04	6.40E+02	<10	>10	4.8E+01	<10	<10	9.3E+04	2.40E+04
18	1.6E+05	9.7E+03	1.5E+05	1.80E+03	<10	>10	3.5E+01	3.3E+01	<10	8.2E+03	2.50E+03
19	2.0E+05	3.2E+03	9.1E+03	3.30E+03	<10	>10	<10	<10	<10	5.0E+03	9.00E+03
20	3.1E+04	4.6E+03	5.4E+03	9.00E+01	<10	>10	3.3E+01	<10	<10	1.9E+02	7.70E+03
21	4.4E+05	8.3E+04	2.0E+05	9.30E+03	<10	>10	1.3E+02	<10	<10	2.2E+04	3.70E+05
22	2.8E+04	3.6E+03	3.5E+03	8.50E+02	<10	>10	1.7E+01	<10	<10	5.9E+03	5.50E+03
23	2.3E+05	1.0E+04	2.4E+04	3.20E+03	<10	>10	3.0E+01	<10	<10	5.7E+03	3.30E+03
24	5.1E+04	1.0E+03	1.9E+03	1.70E+02	1.0E+01	>10	<10	<10	<10	9.1E+02	2.50E+03

*Data of total bacterial count by qPCR were extracted from the publication: Treven, P., A. Mahnic, M. Rupnik, M. Golob, T. Pirs, B. B. Matijasic and P. M. Lorbeg (2019). "Evaluation of Human Milk Microbiota by 16S rRNA Gene Next-Generation Sequencing (NGS) and Cultivation/MALDI-TOF Mass Spectrometry Identification." Front Microbiol 10: 2612

Supplementary table S6: Prevalence of genus-positive samples of raw human milk and of trypsinised MCF10A cells after the adhesion assay.

Genus of the colonies were determined with MALDI-TOF MS. NRID – no reliable edintification.

	**Human milk**	**Adhesion on MCF10A**	
**Genus**	**No. of positive samples (prevalence [%])**	**No. of positive samples (prevalence [%])**	**Adherence ratio**
Staphylococcus	16(100)	14(87,5)	0.88
Propionibacterium	15(93,8)	8(50)	0.53
Corynebacterium	13(81,3)	3(18,8)	0.23
Streptococcus	11(68,8)	4(25)	0.36
Rothia	4(25)	2(12,5)	0.50
Kocuria	3(18,8)	1(6,3)	0.33
Lactobacillus	3(18,8)	0(0,0)	0.00
Acinetobacter	2(12,5)	1(6,3)	0.50
Actinomyces	2(12,5)	1(6,3)	0.50
Escherichia	2(12,5)	0(0,0)	0.00
Gemella	2(12,5)	1(6,3)	0.50
Ochrobactrum	2(12,5)	2(12,5)	1.00
Rhizobium	2(12,5)	0(0,0)	0.00
Bacillus	1(6,3)	0(0,0)	0.00
Bifidobacterium	1(6,3)	0(0,0)	0.00
Enterobacter	1(6,3)	1(6,3)	1.00
Finegoldia	1(6,3)	0(0,0)	0.00
Klebsiella	1(6,3)	1(6,3)	1.00
Pseudomonas	1(6,3)	0(0,0)	0.00
Stenotrophomonas	1(6,3)	2(12,5)	1.50
Veillonella	1(6,3)	0(0,0)	0.00
Delftia	0(0,0)	1(6,3)	na*
Neisseria	0(0,0)	1(6,3)	na*
NRID	15(93,8)	9(56,3)	0.60

**na - not aplicable*

NRID - no reliable identification

Supplementary table S7: Prevalence of species-positive samples of raw human milk and of trypsinised MCF10A cells after the adhesion assay. Species of the colonies were determined with MALDI-TOF MS.

	Human milk		Adhesion on MCF10A		
**Species**	**Prevalence [%]**	**No. of positive samples**	**Prevalence [%]**	**No. of positive samples**	**Adherence ratio**
Staphylococcus epidermidis	100.0	16	87.5	14	0.88
*Cutibacterium acnes**	93.8	15	50.0	8	0.53
Corynebacterium tuberculostearicum	62.5	10	18.8	3	0.30
Staphylococcus hominis	50.0	8	18.8	3	0.38
Streptococcus mitis	43.75	7	12.5	2	0.29
Propionibacterium granulosum	25.0	4	0.0	0	0.00
Staphylococcus warneri	25.0	4	18.8	3	0.75
Streptococcus oralis	25	4	6.3	1	0.25
Streptococcus salivarius	25	4	6.3	1	0.25
Streptococcus vestibularis	25	4	0.0	0	0.00
Corynebacterium simulans	18.8	3	0.0	0	0.00
Rothia mucilaginosa	18.8	3	6.3	1	0.33
Streptococcus parasanguinis	18.75	3	0.0	0	0.00
Corynebacterium kroppenstedtii	12.5	2	6.3	1	0.50
Escherichia coli	12.5	2	0.0	0	0.00
Gemella haemolysans	12.5	2	6.3	1	0.50
Kocuria kristinae	12.5	2	6.3	1	0.50
Lactobacillus rhamnosus	12.5	2	0.0	0	0.00
Ochrobactrum anthropi	12.5	2	12.5	2	1.00
Rhizobium radiobacter	12.5	2	0.0	0	0.00
Staphylococcus aureus	12.5	2	6.3	1	0.50
Staphylococcus capitis	12.5	2	0.0	0	0.00
Staphylococcus haemolyticus	12.5	2	0.0	0	0.00
Staphylococcus lugdunensis	12.5	2	6.3	1	0.50
Streptococcus pneumoniae	12.5	2	0.0	0	0.00
Acinetobacter pittii	6.3	1	6.3	1	1.00
Acinetobacter ursingii	6.3	1	0.0	0	0.00
Actinomyces neuii	6.3	1	6.3	1	1.00

Supplementary table S7 continued

Species	Human milk		Adhesion on MCF10A		
	Prevalence [%]	No. of positive samples	Prevalence [%]	No. of positive samples	Adherence ratio
Actinomyces oris	6.3	1	0.0	0	0.00
Bacillus cereus	6.3	1	0.0	0	0.00
Bifidobacterium breve	6.3	1	0.0	0	0.00
Corynebacterium mucifaciens	6.3	1	0.0	0	0.00
Corynebacterium propinquum	6.3	1	0.0	0	0.00
Corynebacterium striatum	6.3	1	0.0	0	0.00
Enterobacter asburiae	6.3	1	0.0	0	0.00
Enterobacter cloacae	6.3	1	6.3	1	1.00
Finegoldia magna	6.3	1	0.0	0	0.00
Klebsiella oxytoca	6.3	1	6.3	1	1.00
Kocuria rhizophila	6.3	1	0.0	0	0.00
Lactobacillus gasseri	6.3	1	0.0	0	0.00
Pseudomonas monteilii	6.3	1	0.0	0	0.00
Rothia amarae	6.3	1	6.3	1	1.00
Streptococcus parasanguinis	6.3	1	6.3	1	1.00
Staphylococcus pasteuri	6.3	1	6.3	1	1.00
Stenotrophomonas maltophilia	6.25	1	12.5	2	2.00
Streptococcus urinalis	6.25	1	6.3	1	1.00
Veillonella dispar	6.25	1	0.0	0	0.00
Delftia acidovorans	0	0	6.3	1	na**
Neisseria flavescens	0	0	6.3	1	na**

*former *Propionibacterium acnes*

***na - not aplicable*

In vitro model of human MAmmary Gland mIcrobial Colonization (MAGIC) demonstrates distinctive cytokine response to imbalanced human milk microbiota

Primož Treven[1*], Petra Mohar Lorbeg[1], Diana Paveljšek[1], Rok Kostanjšek[2]; Majda Golob[3], Bojana Bogovič Matijašič[1]

Supplementary file 2

List of supplementary figures:

Supplementary Fig. S1:	Growth characteristics of MCF10A cells on porous cell culture membranes (PCCM).
Supplementary Fig. S2:	Relative gene expression for E-cadherin and tight junction proteins during the cell growth on porous cell culture membranes (PCCM).
Supplementary Fig. S3:	Alcian blue/PAS staining of differentiated MCF10A cells grown on porous cell culture membranes (PCCM) for 19 days.
Supplementary Fig. S4:	Relative abundance [%] of each operational taxonomic unit (OTU) per human milk sample in 5 samples obtained by 16S rRNA gene sequencing.
Supplementary Fig. S5	Relative abundance [%] of each bacterial genera per sample in 5 milk samples, determined with cultivation and MALDI-TOF MS identification of selected colonies grown on BHI, TSA, WCA-M and Rogosa.

Supplementary Fig. S1: Growth characteristics of MCF10A cells on porous cell culture membranes (PCCM). **(A)** Transepithelial electrical resistance (TEER) development in relation to frequency of media change (every day-1-DAY *vs* every two days-2-DAY) and in relation to cell manipulation at room temperature (RT) or at 37 °C by using heating pad (HP). **(B)** Relation between sodium fluorescein flux and TEER during cell growth on PCCM. Cells reached the highest TEER in 14-17 days after seeding on PCCM (the experiment was performed without heating pad). The experiments were performed in 4 biological replicates. Results are shown as mean ± standard error.

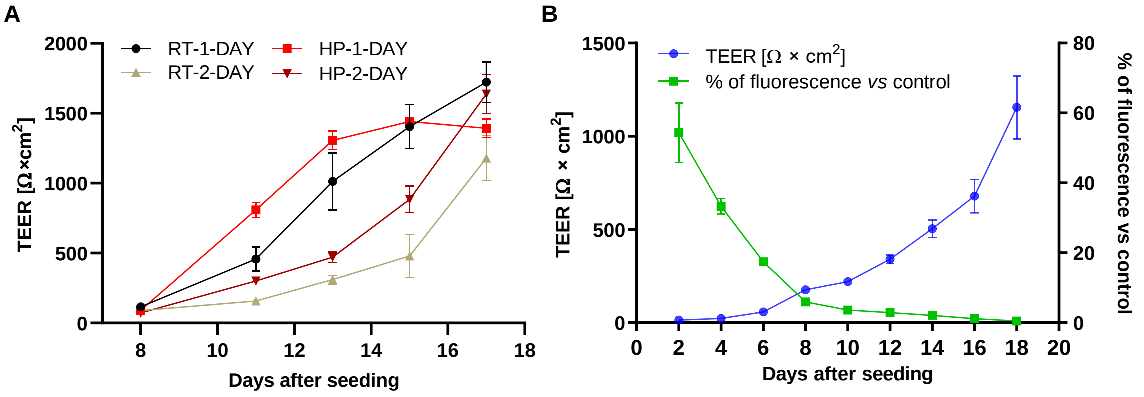

Supplementary Fig. S2: Relative gene expression for E-cadherin and tight junction proteins during the cell growth on porous cell culture membranes (PCCM). Results are shown as normalised gene expression relative to the expression in cells confluent on 48-well cell culture plates (CCP). Statistical significance was analyzed with non-parametric one-way ANOVA (Kruskal-Wallis statistic) and Dunn's multiple comparison test with adjustments of p-values to account for multiple comparisons; *p<0.05, **p<0.01. The experiment was performed in three independent experiments with two biological and two technical replicates. Dotted line marks two-fold change in gene expression.

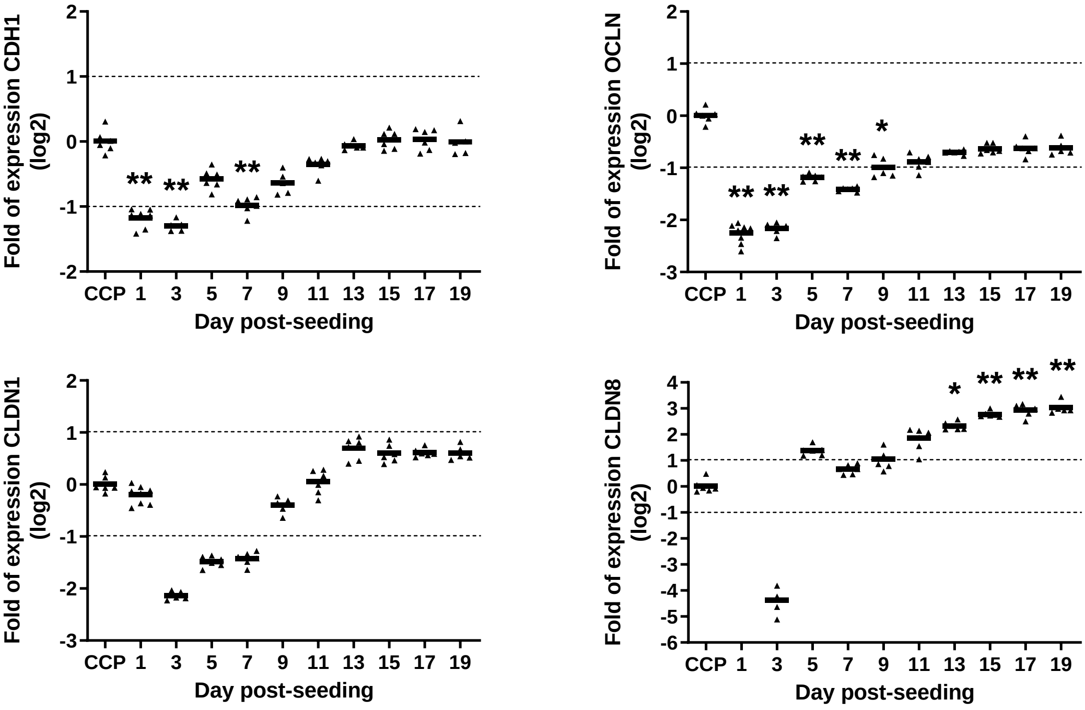

Supplementary Fig. S3: Alcian blue/PAS staining of differentiated MCF10A cells grown on porous cell culture membranes (PCCM) for 19 days. Cells grown on PCCM were washed once with HBSS and fixed in 4% paraformaldehyde. After dehydration in series of ethanol concentrations, the inserts were embedded in paraffin, sectioned, mounted on glass slides, and allowed to dry. Two µm thick paraffin tissue sections were stained with Alcian blue at pH 2.5 (A and B) and Periodic-Acid Schiff (C and D) according to the protocols of the Institute of Patology, Wild Animals, Fish and Bees of the Veterinary Faculty University of Ljubljana, covered and examined by light microscopy.

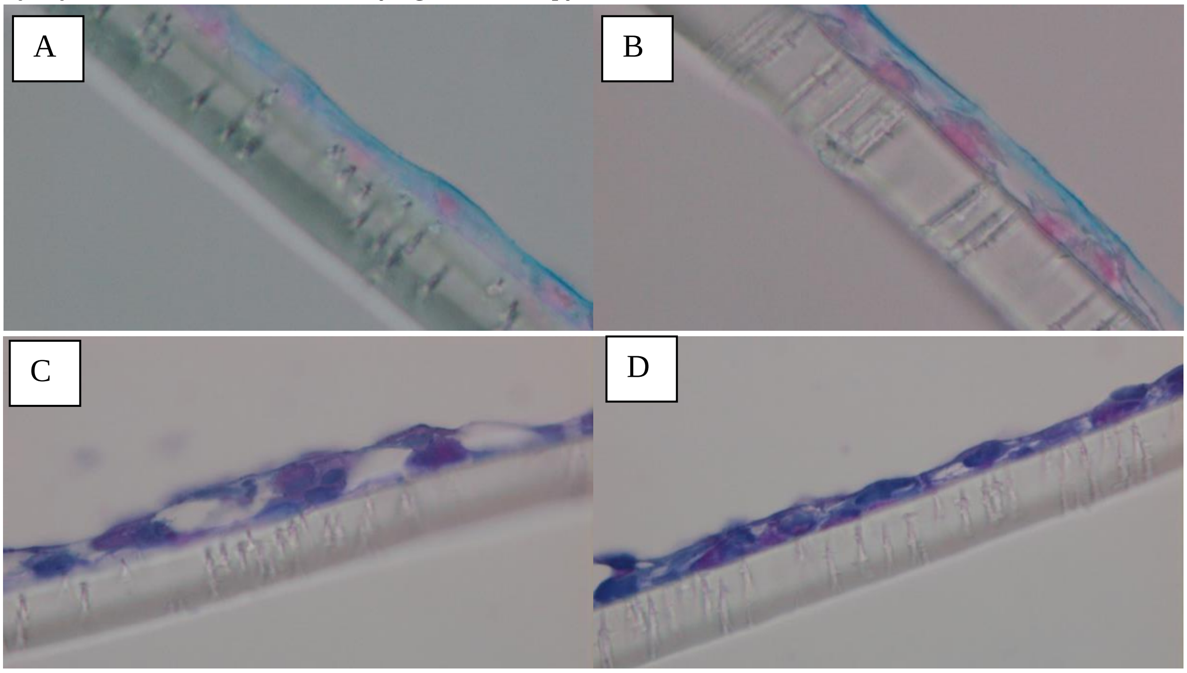

Supplementary Fig. S4: Relative abundance [%] of each operational taxonomic unit (OTU) per human milk sample in 5 samples obtained by 16S rRNA gene sequencing.*
Each sample was subsampled to 10000 reads/sample. Taxonomy is inferred with RDP reference base (mothur, trainset16). OTUs that included less than 0.01 % of total reads obtained were removed. Due to clarity, only OTUs represented in more than 5 % in at least one sample are presented while others are summed as “Others”.

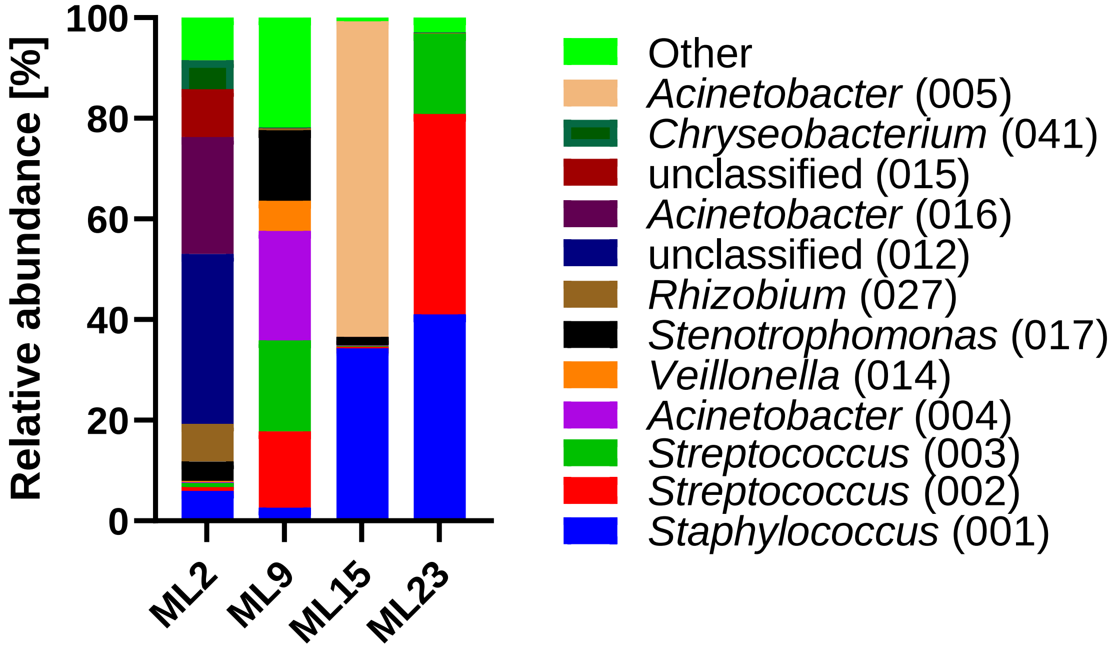

*Data extracted from the publication: Treven, P., A. Mahnic, M. Rupnik, M. Golob, T. Pirs, B. B. Matijasic and P. M. Lorbeg (2019). "Evaluation of Human Milk Microbiota by 16S rRNA Gene Next-Generation Sequencing (NGS) and Cultivation/MALDI-TOF Mass Spectrometry Identification." Front Microbiol 10: 2612

Supplementary Fig. S5: Relative abundance [%] of each bacterial genera per sample in 5 milk samples, determined with cultivation and MALDI-TOF MS identification of selected colonies grown on BHI, TSA, WCA-M and Rogosa.* Due to clarity, only genera represented in more than 5 % in at least one sample are presented while the rest are summed as "Others and NRID (no reliable identification).

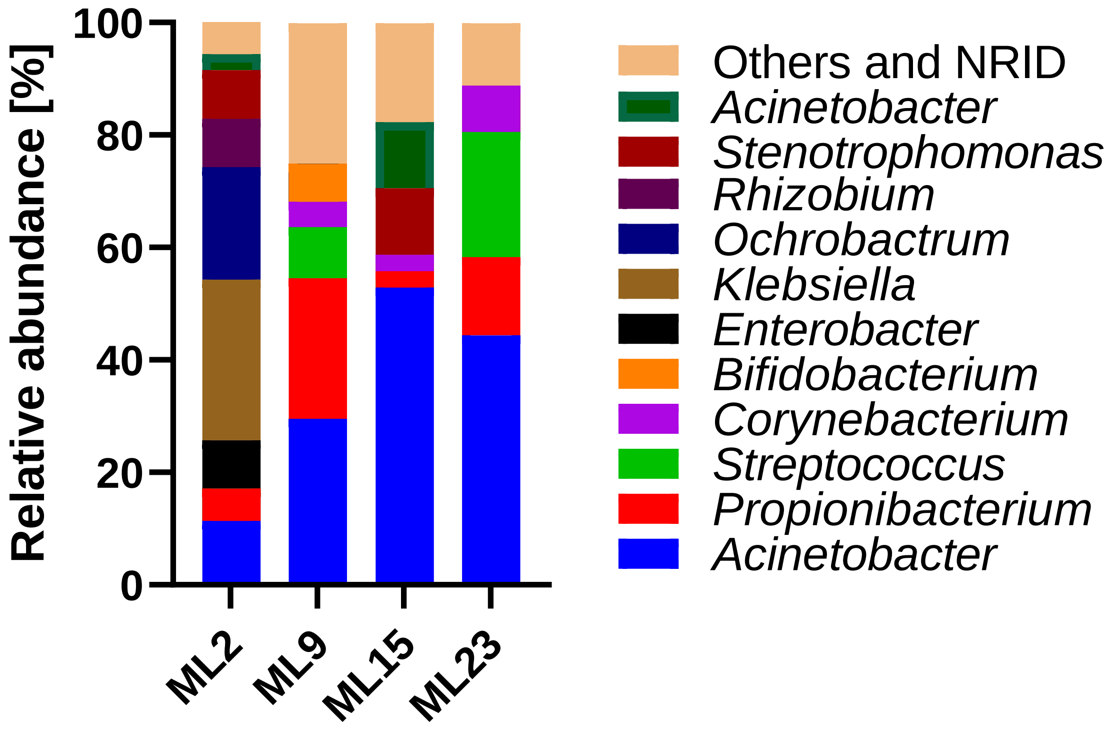

*Data extracted from the publication: Treven, P., A. Mahnic, M. Rupnik, M. Golob, T. Pirs, B. B. Matijasic and P. M. Lorbeg (2019). "Evaluation of Human Milk Microbiota by 16S rRNA Gene Next-Generation Sequencing (NGS) and Cultivation/MALDI-TOF Mass Spectrometry Identification." Front Microbiol 10: 26
